# Broad host range of SARS-CoV-2 predicted by comparative and structural analysis of ACE2 in vertebrates

**DOI:** 10.1073/pnas.2010146117

**Published:** 2020-08-21

**Authors:** Joana Damas, Graham M. Hughes, Kathleen C. Keough, Corrie A. Painter, Nicole S. Persky, Marco Corbo, Michael Hiller, Klaus-Peter Koepfli, Andreas R. Pfenning, Huabin Zhao, Diane P. Genereux, Ross Swofford, Katherine S. Pollard, Oliver A. Ryder, Martin T. Nweeia, Kerstin Lindblad-Toh, Emma C. Teeling, Elinor K. Karlsson, Harris A. Lewin

**Affiliations:** ^a^The Genome Center, University of California, Davis, CA 95616;; ^b^School of Biology and Environmental Science, University College Dublin, Belfield, Dublin 4, Ireland;; ^c^Graduate Program in Pharmaceutical Sciences and Pharmacogenomics, Quantitative Biosciences Consortium, University of California, San Francisco, CA 94117;; ^d^Gladstone Institute of Data Science and Biotechnology, San Francisco, CA 94158;; ^e^Cancer Program, Broad Institute of MIT and Harvard, Cambridge, MA 02142;; ^f^Genetic Perturbation Platform, Broad Institute of MIT and Harvard, Cambridge, MA 02142;; ^g^Max Planck Institute of Molecular Cell Biology and Genetics, 01307 Dresden, Germany;; ^h^Max Planck Institute for the Physics of Complex Systems, 01187 Dresden, Germany;; ^i^Center for Systems Biology Dresden, 01307 Dresden, Germany;; ^j^Center for Species Survival, Smithsonian Conservation Biology Institute, National Zoological Park, Front Royal, VA 22630;; ^k^Department of Computational Biology, School of Computer Science, Carnegie Mellon University, Pittsburgh, PA 15213;; ^l^Department of Ecology, Tibetan Centre for Ecology and Conservation at WHU-TU, Hubei Key Laboratory of Cell Homeostasis, College of Life Sciences, Wuhan University, Wuhan 430072, China;; ^m^College of Science, Tibet University, Lhasa 850000, China;; ^n^Broad Institute of MIT and Harvard, Cambridge, MA 02142;; ^o^Department of Epidemiology & Biostatistics, Institute for Computational Health Sciences, and Institute for Human Genetics, University of California, San Francisco, CA 94158;; ^p^Chan Zuckerberg Biohub, San Francisco, CA 94158;; ^q^San Diego Zoo Institute for Conservation Research, Escondido, CA 92027;; ^r^Department of Evolution, Behavior, and Ecology, Division of Biology, University of California San Diego, La Jolla, CA 92093;; ^s^Department of Restorative Dentistry and Biomaterials Sciences, Harvard School of Dental Medicine, Boston, MA 02115;; ^t^School of Dental Medicine, Case Western Reserve University, Cleveland, OH 44106;; ^u^Marine Mammal Program, Department of Vertebrate Zoology, Smithsonian Institution, Washington, DC 20002;; ^v^Science for Life Laboratory, Department of Medical Biochemistry and Microbiology, Uppsala University, 751 23 Uppsala, Sweden;; ^w^Bioinformatics and Integrative Biology, University of Massachusetts Medical School, Worcester, MA 01655;; ^x^Program in Molecular Medicine, University of Massachusetts Medical School, Worcester, MA 01655;; ^y^Department of Evolution and Ecology, University of California, Davis, CA 95616;; ^z^John Muir Institute for the Environment, University of California, Davis, CA 95616

**Keywords:** SARS-CoV-2, COVID-19, ACE2, comparative genomics, species conservation

## Abstract

The novel coronavirus severe acute respiratory syndrome coronavirus 2 (SARS-CoV-2) is the cause of COVID-19. The main receptor of SARS-CoV-2, angiotensin I converting enzyme 2 (ACE2), is now undergoing extensive scrutiny to understand the routes of transmission and sensitivity in different species. Here, we utilized a unique dataset of ACE2 sequences from 410 vertebrate species, including 252 mammals, to study the conservation of ACE2 and its potential to be used as a receptor by SARS-CoV-2. We designed a five-category binding score based on the conservation properties of 25 amino acids important for the binding between ACE2 and the SARS-CoV-2 spike protein. Only mammals fell into the medium to very high categories and only catarrhine primates into the very high category, suggesting that they are at high risk for SARS-CoV-2 infection. We employed a protein structural analysis to qualitatively assess whether amino acid changes at variable residues would be likely to disrupt ACE2/SARS-CoV-2 spike protein binding and found the number of predicted unfavorable changes significantly correlated with the binding score. Extending this analysis to human population data, we found only rare (frequency <0.001) variants in 10/25 binding sites. In addition, we found significant signals of selection and accelerated evolution in the ACE2 coding sequence across all mammals, and specific to the bat lineage. Our results, if confirmed by additional experimental data, may lead to the identification of intermediate host species for SARS-CoV-2, guide the selection of animal models of COVID-19, and assist the conservation of animals both in native habitats and in human care.

## Significance

The novel severe acute respiratory syndrome coronavirus 2 (SARS-CoV-2) is the cause of COVID-19, a major pandemic that threatens millions of human lives and the global economy. We identified a large number of mammals that can potentially be infected by SARS-CoV-2 via their ACE2 proteins. This can assist the identification of intermediate hosts for SARS-CoV-2 and hence reduce the opportunity for a future outbreak of COVID-19. Among the species we found with the highest risk for SARS-CoV-2 infection are wildlife and endangered species. These species represent an opportunity for spillover of SARS-CoV-2 from humans to other susceptible animals. Given the limited infectivity data for the species studied, we urge caution not to overinterpret the predictions of the present study.

Severe acute respiratory syndrome coronavirus 2 (SARS-CoV-2) is the cause of COVID-19, a major pandemic that threatens millions of lives and the global economy (1). Comparative analysis of SARS-CoV-2 and related coronavirus sequences has shown that SARS-CoV and SARS-CoV-2 likely had ancestors that originated in bats, followed by transmission to an intermediate host, and that both viruses may have an extended host range that includes primates and other mammals (1–3). Many mammalian species host coronaviruses and these infections are frequently associated with severe clinical diseases, such as respiratory and enteric disease in pigs and cattle (4, 5). Molecular phylogenetics revealed that at least one human coronavirus (HCov-OC43) may have originated in cattle or swine and that this virus was associated with a human pandemic that emerged in the late 19th century (6). Recent data indicate that coronaviruses can be transmitted from bats to other wildlife species and humans (7), and from humans to tigers (8) and pigs (9). Therefore, understanding the host range of SARS-CoV-2 and related coronaviruses is essential for improving our ability to predict and control future pandemics. It is also crucial for protecting populations of wildlife species in native habitats and under human care, particularly nonhuman primates, which may be susceptible to COVID-19 (10).

The angiotensin I converting enzyme 2 (ACE2) serves as a functional receptor for the spike protein (S) of SARS-CoV and SARS-CoV-2 (11, 12). Under normal physiological conditions, ACE2 is a dipeptidyl carboxypeptidase that catalyzes the conversion of angiotensin I into angiotensin 1-9, a peptide of unknown function (13). ACE2 also converts angiotensin II, a vasoconstrictor, into angiotensin 1-7, a vasodilator that affects the cardiovascular system (13) and may regulate other components of the renin–angiotensin system (14). The host range of SARS-CoV-2 may be extremely broad due to the conservation of ACE2 in mammals (2, 12). While SARS-CoV-2 and related coronaviruses use human ACE2 as a primary receptor, coronaviruses may use other proteases as receptors, such as CD26 (DPP4) for Middle East Respiratory Syndrome (MERS)-CoV (15), thus limiting or extending host range.

In humans, ACE2 may be a cell membrane protein or it may be secreted (13). The secreted form is created primarily by enzymatic cleavage of surface-bound ACE2 by ADAM17 and other proteases (13). *ACE2* maps to the human X chromosome. Many synonymous and nonsynonymous mutations have been identified in this gene, although most of these are rare at the population level (16), and few are believed to affect cellular susceptibility to human coronavirus infections (17). Site-directed mutagenesis and coprecipitation of SARS-CoV constructs have revealed critical residues on the ACE2 tertiary structure that are essential for binding to the virus receptor-binding domain (RBD) (18). These findings are supported by the cocrystallization and structural determination of the SARS-CoV and SARS-CoV-2 S proteins with human ACE2 (12, 19, 20), as well as binding affinity with nonhuman ACE2 (18). Coronaviruses may adapt to new hosts in part through mutations in S that enhance binding affinity for ACE2. The best-studied example is the evolution of SARS-CoV-like coronaviruses in the masked palm civet, which is believed to be the intermediate host for transmission of a SARS-CoV-like virus from bats to humans (2). The masked palm civet SARS-CoV S acquired two mutations that increased its affinity for human ACE2 (2). An intermediate host for SARS-CoV-2 has not been identified definitively, although the Malayan pangolin has been proposed (21).

Comparative analysis of ACE2 protein sequences can be used to predict their ability to bind SARS-CoV-2 S (2) and therefore may yield important insights into the biology and potential zoonotic transmission of SARS-CoV-2 infection. Recent work predicted ACE2/SARS-CoV-2 S-binding affinity in some vertebrate species, but phylogenetic sampling was extremely limited (10, 22). Here, we used a combination of comparative genomic approaches and protein structural analysis to assess the potential of ACE2 homologs from 410 vertebrate species (including representatives from all vertebrate classes: fishes, amphibians, birds, reptiles, and mammals) to serve as a receptor for SARS-CoV-2 and to understand the evolution of ACE2/SARS-CoV-2 S-binding sites. Our results reinforce earlier findings on the natural host range of SARS-CoV-2 and predict a broader group of species that may serve as a reservoir or intermediate host(s) for this virus. Importantly, many threatened and endangered species were found to be at potential risk for SARS-CoV-2 infection based on their ACE2 binding score, suggesting that as the pandemic spreads humans could inadvertently introduce a potentially devastating new threat to these already vulnerable populations, especially the great apes and other primates.

## Results

### Comparison of Vertebrate ACE2 Sequences and Their Predicted Ability to Bind SARS-CoV-2.

**Fig. 1.**
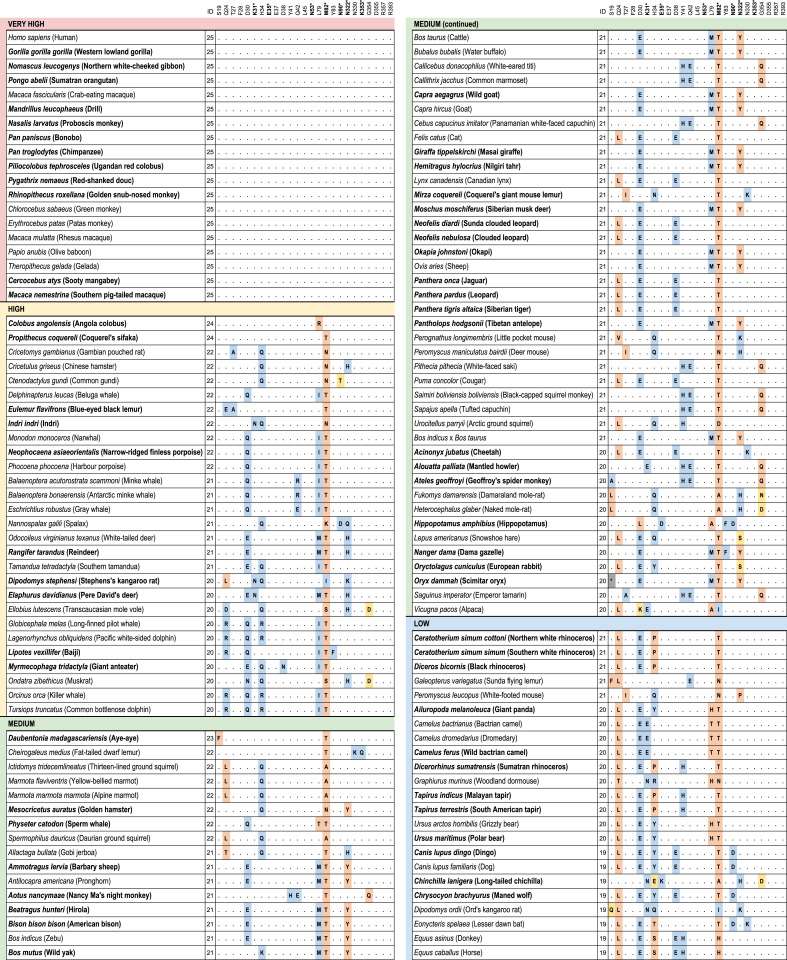
Cross-species conservation of ACE2 at the known binding residues and predictions of SARS-CoV-2 S-binding propensity. Species are sorted by binding scores. The ID column depicts the number of amino acids identical to human binding residues. Bold amino acid positions (also labeled with asterisks) represent residues at binding hot spots and constrained in the scoring scheme. Each amino acid substitution is colored according to its classification as nonconservative (orange), semiconservative (yellow), or conservative (blue), as compared to the human residue. Bold species names depict species with threatened IUCN risk status. The 410 vertebrate species dataset is available in Dataset S1.

**Fig. 2.**
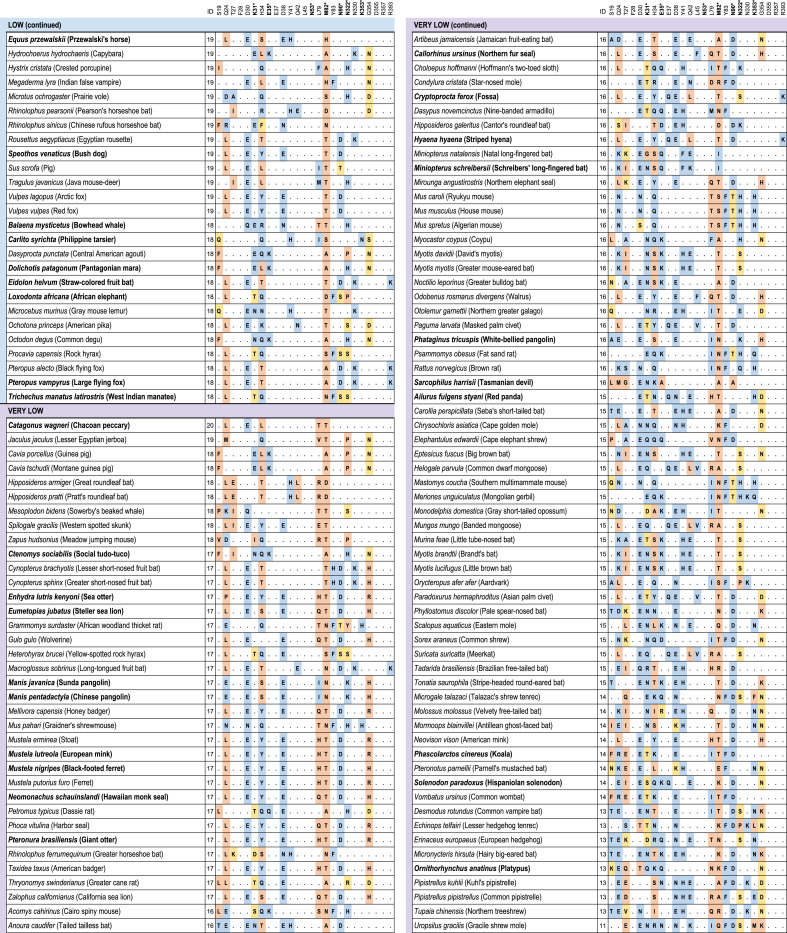
Cross-species conservation of ACE2 at the known binding residues and predictions of SARS-CoV-2 S-binding propensity. Species are sorted by binding scores. The ID column depicts the number of amino acids identical to human binding residues. Bold amino acid positions (also labeled with asterisks) represent residues at binding hot spots and constrained in the scoring scheme. Each amino acid substitution is colored according to its classification as nonconservative (orange), semiconservative (yellow), or conservative (blue), as compared to the human residue. Bold species names depict species with threatened IUCN risk status. The 410 vertebrate species dataset is available in Dataset S1.

We identified 410 unique vertebrate species with *ACE2* orthologs (Dataset S1), including representatives of all vertebrate taxonomic classes. Among these were 252 mammals, 72 birds, 65 fishes, 17 reptiles, and 4 amphibians. Twenty-five amino acids corresponding to known SARS-CoV-2 S-binding residues (10, 12, 20) were examined for their similarity to the residues in human ACE2 (Figs. 1 and 2 and Dataset S1). On the basis of known interactions between specific residues on ACE2 and the RBD of SARS-CoV-2 S, a set of rules was developed for predicting the propensity for S binding to ACE2 from each species (*Materials and Methods*). Five score categories were predicted: very high, high, medium, low, and very low. Results for all species are shown in Dataset S1, and results for mammals only are shown in Figs. 1 and 2. The very high classification had at least 23/25 ACE2 residues identical to human ACE2 and other constraints at SARS-CoV-2 S-binding hot spots (*Materials and Methods*). The 18 species predicted as very high were all Old-World primates and great apes with ACE2 proteins identical to human ACE2 across all 25 binding residues. The ACE2 proteins of 28 species were classified as having a high propensity for binding the SARS-CoV-2 S RBD. Among them are 12 cetaceans (whales and dolphins), 7 rodents, 3 cervids (deer), 3 lemuriform primates, 2 representatives of the order Pilosa (giant anteater and southern tamandua), and 1 Old-World primate (Angola colobus; Fig. 1). Fifty-seven species scored as medium for the propensity of their ACE2 to bind SARS-CoV-2 S. This category has at least 20/25 residues identical to human ACE2 but more relaxed constraints for critical binding residues. All species with medium score are mammals distributed across six orders.

Among Carnivora, 9/43 scored medium, 9/43 scored low, and 25/43 scored very low (Figs. 1 and 2). The carnivores scoring medium were exclusively felids, including the domestic cat and Siberian tiger. Among the 13 primate species scoring medium*,* there were 10 New-World primates and three lemurs. Of 45 rodent species, 11 scored medium. Twenty-one of 30 artiodactyls scored medium, including several important wild and domesticated ruminants, such as domesticated cattle, bison, sheep, goat, water buffalo, Masai giraffe, and Tibetan antelope. Species scoring medium also included two of three lagomorphs and one cetacean.

All chiropterans (bats) scored low (*n* = 8) or very low (*n* = 29; Fig. 2), including the Chinese rufous horseshoe bat, from which a coronavirus (SARSr-CoV ZC45) related to SARS-CoV-2 was identified (1). Only 7.7% (3/39) primate species’ ACE2 scored low or very low, and 61% of rodent species scored low (10/46) or very low (18/46). All monotremes (*n* = 1) and marsupials (*n* = 4), birds (*n* = 72), fish (*n* = 65), amphibians (*n* = 4), and reptiles (*n* = 17) scored very low, with fewer than 18/25 ACE2 residues identical to the human and many nonconservative amino acid substitutions at the remaining nonidentical sites (Dataset S1). Notable species scoring very low include the Chinese pangolin, Sunda pangolin, and white-bellied pangolin (Fig. 2 and Dataset S1).

### Structural Analysis of the ACE2/SARS-CoV-2 S-Binding Interface.

**Fig. 3.**
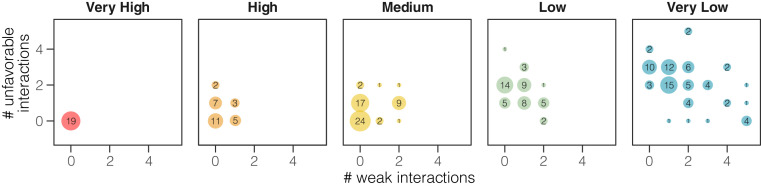
Congruence between binding score and the structural homology analysis. Species predicted with very high (red) or high binding scores (orange) have significantly fewer amino acid substitutions rated as potentially altering the binding interface between ACE2 and SARS-CoV-2 using protein structural analysis when compared to species with low (green) or very low (blue) binding scores. The more severe unfavorable variants are counted on the *y* axis and less severe weaken variants on the *x* axis. Black numerical labels indicate species count.

We complemented the sequence identity-based scoring scheme with a qualitative structure-based scoring system. Our approach was to take the 55 variants of individual residues observed in the ACE2 binding interface, excluding glycosylation sites, from 28 representative species, and identify the best-fit rotamer for each variant when modeled onto the human crystal structure 6MOJ (12). Each variant was then assigned to one of three groups: neutral (likely to maintain similar contacts; 18 substitutions), weaken (likely to weaken the interaction; 14 substitutions), or unfavorable (likely to introduce unfavorable interactions; 23 substitutions; *SI Appendix*, Fig. S1). Variations of residue S19 were excluded because of conflicting results between the two structures of the human ACE2/SARS-CoV-2 S protein complexes 6MOJ and 6VW1 at this site (the two structures were in agreement for all other residues at the binding interface). The structural binding assessments complement the sequence identity analysis, with the fraction of residues ranked as unfavorable correlating very strongly with the substitution scoring scheme (Spearman correlation rho = 0.76; *P* < 2.2e-16; Fig. 3). To check for easily identifiable gross conformational changes between ACE2 proteins of different species that could potentially cause misinterpretation of the ACE2/SARS-CoV-2 S interface, we also generated homology models of ACE2 from the 28 representative species and compared them to the human structures. All models showed high similarity to the human protein along the C⍺ backbone (*SI Appendix*, Fig. S2) with an rmsd range of 0.06 to 0.17. Among all 28 structures, high coverage ranging from 91 to 99% and high global model quality estimation ranging between 0.82 and 0.89 (*SI Appendix*, Table S1), as assessed in CHIMERA, indicated a lack of major conformational changes between species and supported the validity of using human structures as a template for modeling variants of ACE2 interface residues across species.

### Structural Analysis of Variation in Human *ACE2*.

We examined the variation in ACE2 binding residues within humans, some of which have been proposed to alter binding affinity (17, 23–26). We integrated data from six different sources, dbSNP, 1KGP, Topmed, UK10K, gnomAD, and CHINAMAP, and identified a total of 11 variants in 10 of the 25 ACE2 binding residues (Dataset S2). All variants found are rare, with allele frequency (*f*) < 0.01 in any individual population and *f* < 0.0007 across all populations. Three of the 11 single-nucleotide variants were silent, leading to synonymous amino acid changes, seven were missense variants resulting in conservative amino acid substitutions, and one, S19P, resulted in a semiconservative substitution. S19P has the highest allele frequency of the 11 variants, with *f =* 0.0003 across all populations (16). We evaluated, by structural homology, six missense variants. Four were neutral and two weakening (E35K, *f =* 0.000016; E35D, *f =* 0.000279799). S19P was not included in our structural homology assessment, but a recent study predicted it would increase ACE2/SARS-CoV-2 binding affinity (27). Thus, with an estimated summed frequency of 0.001 (maximum of 0.004 in any single population), genetic variation in the human ACE2/SARS-CoV-2 S-binding interface is rare overall, and it is unclear whether the existing variation increases or decreases susceptibility to infection.

### Evolution of ACE2 across Mammals.

We next investigated the evolution of ACE2 variation in vertebrates, including how patterns of positive selection compare between bats, a mammalian lineage that harbors a high diversity of coronaviruses (28), and other mammalian clades. We first inferred the phylogeny of *ACE2* using our 410-vertebrate alignment and IQTREE, using the best-fit model of sequence evolution (JTT+F + R7) and rooting the topology on fishes (Dataset S3 and *SI Appendix*, Fig. S3). We then assayed sequence conservation with phyloP. The majority of ACE2 codons are significantly conserved across vertebrates and across mammals (Dataset S4.1), likely reflecting its critical function in the renin–angiotensin system (29). Ten residues in the ACE2 binding domain are exceptionally conserved in Chiroptera and/or Rodentia (Dataset S4.2).

We next used phyloP and CodeML to test for accelerated sequence evolution and positive selection, respectively. PhyloP compares the rate of evolution at each codon to the expected rate in a model estimated from third nucleotide positions of the codon and is agnostic to synonymous versus nonsynonymous substitutions (dN/dS). CodeML uses ⍵ = dN/dS > 1 and Bayes empirical Bayes (BEB) scores to identify codons under positive selection and was run on a subset of 64 representative mammals (*Materials and Methods*). In this way, PhyloP identifies residues evolving at a rate higher than the estimated neutral rate of evolution. In addition, CodeML identifies residues exhibiting an excess of nonsynonymous over synonymous substitutions.

**Fig. 4.**
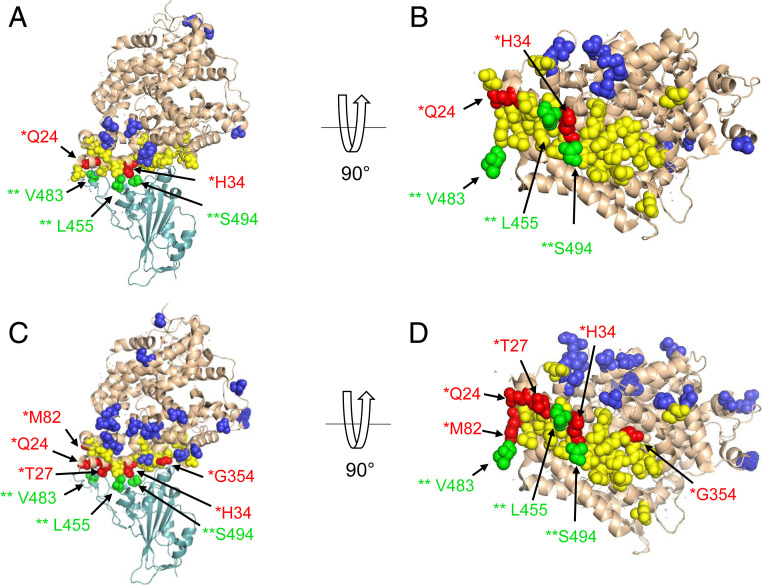
Residues at the binding interface between ACE2 and SARS-CoV-2 S are under positive selection (CodeML analysis). In the SARS-CoV-2 spike protein RBD (light teal), this includes three positively selected residues (green, labeled with two asterisks). In ACE2 (wheat-colored, with binding interface residues in yellow), selected residues occur both outside the binding interface (dark blue) and inside the binding interface (red, labeled with one asterisk). (*A*) Positively selected residues in all mammals, including two at the binding interface. (*B*) *A* with 90° rotation. (*C*) Positively selected residues in the Chiroptera lineage, including five at the binding interface. (*D*) *C* with 90° rotation.

*ACE2* shows significant evidence of positive selection across mammals (⍵ = 1.83, likelihood ratio test [LRT] = 194.13, *P* < 0.001; Datasets S4.3 and S4.4). Almost 10% of codons (*n* = 73; 9 near the binding interface) are accelerated within mammals (Datasets S4.1 and S4.5), and 18 of these have BEB scores greater than 0.95, indicating positively selected residues (Datasets S4.5 and S4.6 and *SI Appendix*, Fig. S4). Nineteen accelerated residues, including two positively selected codons (Q24 and H34), are known to interact with SARS-CoV-2 S (Fig. 4 *A* and *B*, Dataset S4.5, and *SI Appendix*, Fig. S5). Q24 has not been observed to be polymorphic within the human population, and H34 harbors a synonymous polymorphism (*f* = 0.00063) but no nonsynonymous polymorphisms (Dataset S2).

This pattern of acceleration and positive selection in *ACE2* also holds for individual mammalian lineages. Using CodeML, positive selection was detected within the orders Chiroptera (LRT = 346.40, ⍵ = 3.44, *P* < 0.001), Cetartiodactyla (LRT = 92.86, ⍵ = 3.83, *P* < 0.001), Carnivora (LRT = 65.66, ⍵ = 2.27, *P* < 0.001), Primates (LRT = 72.33, ⍵ = 3.16, *P* < 0.001), and Rodentia (LRT = 91.26, ⍵ = 1.77, *P* < 0.001). Overall, bats had more positively selected sites with significant BEB scores (29 sites in Chiroptera compared to 10, 8, 7, and 15 sites in Cetartiodactyla, Carnivora, Primates, and Rodentia, respectively). Positive selection was found at multiple ACE2/SARS-CoV-2 S-binding residues in the bat-specific alignment. Parameters inferred by CodeML were consistent across different models of evolution (Dataset S4.6). PhyloP was used to assess shifts in the evolutionary rate within mammalian lineages, for each assessing signal relative to a neutral model trained on species from the specified lineage (Datasets S4.7–S4.12 and *SI Appendix*, Fig. S6). We discovered six binding residues that are accelerated in one or more of Chiroptera, Rodentia, or Carnivora, five of which also showed evidence for positive selection; G354 was accelerated in all of these lineages (Dataset S4.13).

Given pervasive signatures of adaptive evolution in *ACE2* across mammals, we next sought to test if *ACE2* in any mammalian lineages is evolving particularly rapidly compared to the others. CodeML branch-site tests identified positive selection in both the ancestral Chiroptera branch (one amino acid, ⍵ = 26.7, LRT = 4.22, *P* = 0.039) and ancestral Cetartiodactyla branch (two amino acids, ⍵ = 10.38, LRT = 7.89, *P* = 0.004; Dataset S4.3) using 64 mammals. These residues did not correspond to known viral binding sites. We found no evidence for lineage-specific positive selection in the ancestral primate, rodent, or carnivore lineages. PhyloP identified lineage-specific acceleration in Chiroptera, Carnivora, Rodentia, Artiodactyla, and Cetacea relative to mammals (Datasets S4.14–S4.18 and *SI Appendix*, Fig. S7). The power to detect acceleration within a clade scaled with the branch length of the subtree, with rodents having the highest and bats the second-highest amount of power (*SI Appendix*, Fig. S8 and Table S2). Bats have a particularly high level of accelerated evolution (18 codons; *P* < 0.05). Of these accelerated residues, T27 and M82 are binding residues for SARS-CoV-2 S, with some bat subgroups having amino acid substitutions predicted to lead to less favorable binding of SARS-CoV-2 (Fig. 4 *C* and *D* and *SI Appendix*, Fig. S1). Surprisingly, a residue that is conserved overall in our 410 species alignment and in the mammalian subset, Q728, is perfectly conserved in all 37 species of bats except for Old-World fruit bat species (Pteropodidae; *n* = 8), which have a substitution from Q to E. These results support the theory that *ACE2* is under lineage-specific selective pressures in bats relative to other mammals.

### Positive Selection in SARS-CoV-2 S Protein.

Positive selection was found across 43 viral strains (Dataset S4.19) at sites L455, V483, and S494 in the SARS-CoV-2 S sequence using CodeML (⍵ = 2.78, LRT = 93.72, *P* < 0.001). All of these sites lie within or near the ACE2/SARS-CoV-2 S RBD binding sites (Fig. 4).

## Discussion

Phylogenetic analysis of coronaviruses has demonstrated that the immediate ancestor of SARS-CoV-2 most likely originated in a bat species (1). However, whether SARS-CoV-2 or the progenitor of this virus was transmitted directly to humans or through an intermediate host is not yet resolved. To identify candidate intermediate host species and species at risk for SARS-CoV-2 infection, we undertook a deep comparative genomic, evolutionary, and structural analysis of ACE2, which serves as the SARS-CoV-2 receptor in humans. We drew on the rapidly growing database of annotated vertebrate genomes, including new genomes produced by the Genomes 10K-affiliated Bat1K Consortium, Zoonomia, and Vertebrate Genomes Project, and other sources (30, 31). We conducted a phylogenetic analysis of ACE2 orthologs from 410 vertebrate species and predicted their propensity to bind the SARS-CoV-2 S using a score based on amino acid substitutions at 25 consensus human ACE2 binding residues (12, 20). Similarity-based methods are frequently used for predicting cross-species transmission of viruses (32, 33), including SARS-CoV (2). We supported these predictions with comprehensive structural analysis of the ACE2 binding site complexed with SARS-CoV-2 S. We also tested the hypothesis that the ACE2 receptor is under selective constraints in mammalian lineages with different susceptibilities to coronaviruses.

We predict that species scoring as very high and high for propensity of SARS-CoV-2 S binding to ACE2 will have a high probability of becoming infected by the virus and thus may be potential intermediate hosts for virus transmission. We also predict that many species having a medium score have some risk of infection, and species scored as very low and low are less likely to be infected by SARS-CoV-2 via the ACE2 receptor. Importantly, our predictions are based solely on in silico analyses and must be confirmed by direct experimental data. The prediction accuracy of the model may be improved in the future as more extensive data are generated showing the impact of ACE2 mutations on its binding affinity for SARS-CoV-2 S, which may enable knowledge-based weighting of residues in the scoring algorithm. Until the present model’s accuracy can be confirmed with additional experimental data, we urge caution not to overinterpret the predictions of the present study. This is especially important with regards to species, endangered or otherwise, in human care. While species ranked high or medium may be susceptible to infection based on the features of their ACE2 residues, pathological outcomes may be very different among species depending on other mechanisms, such as immune response, that could affect virus replication and spread to target cells, tissues, and organs. Furthermore, we cannot exclude the possibility that infection in any species occurs via another cellular receptor (for a review see ref. 34), as shown for other betacoronaviruses (35), or lower-affinity interactions with ACE2 as proposed for SARS-CoV (2). Nonetheless, our predictions provide a useful starting point for the selection of appropriate animal models for COVID-19 research and identification of species that may be at risk for human-to-animal or animal-to-animal transmissions of SARS-CoV-2.

Several recent studies examined the role of ACE2 in SARS-CoV-2 binding and cellular infection and its relationship to experimental and natural infections in different species (26, 35–40). Our study design differs substantially from those in several aspects: 1) we analyzed a larger number of primates, carnivores, rodents, cetartiodactyls, and other mammalian orders and an extensive phylogenetic sampling of fishes, birds, amphibians, and reptiles; 2) we analyzed the full set of S-binding residues across the ACE2 binding site, which was based on a consensus set from two independent studies (12, 20); 3) we used different methodologies to assess ACE2 binding capacity for SARS-CoV-2 S; and 4) our study tested for selection and accelerated evolution across the entire ACE2 protein. While our results are consistent with the results and conclusions of Melin et al. (38) on the predicted susceptibility of primates to SARS-CoV-2, particularly Old-World primates, we made predictions for a larger number of primates (*n* = 39 vs. *n* = 27), bats (*n* = 37 vs. *n* = 7), other mammals (*n* = 176 vs. *n* = 5), and other vertebrates (*n* = 158 vs. *n* = 0). When ACE2 from species in our study were compared with results of other studies there were many consistencies, such as the low risk for rodents, but some predictions differ, such as the relatively high risk predicted by others for SARS-CoV-2 S binding in pangolin and horse (39), civet (40), Chinese rufous horseshoe bat (40), and turtles (22). Our results are generally consistent with a study that tested binding affinity of soluble ACE2 for the SARS-CoV-2 S RBD using saturation mutagenesis (27), particularly in the binding hot-spot region of ACE2 residues 353 to 357 (*SI Appendix*, Fig. S1). Importantly, as compared with other studies, our results greatly expanded the number of candidate intermediate hosts and identified many additional threatened species that could be at risk for SARS-CoV-2 infection via their ACE2 receptors.

### Evolution of *ACE2*.

Variation in *ACE2* in the human population is rare (16). Overall, ACE2 is intolerant of loss-of-function mutations [pLI = 0.998; LOEUF = 0.25 in gnomAD v2.1.1 (16)]. We examined a large set of ACE2 variants for their potential differences in binding to SARS-CoV-2 S and their relationship to selected and accelerated sites. We found rare coding variants that would result in missense mutations causing substitutions in 7/25 binding residues (Dataset S2). Some of those [e.g., E35K, *f* = 0.00001636 (16)] could reduce the virus binding affinity as per our structural analysis (Dataset S2) but would potentially lower the susceptibility to the virus only in a very small fraction of the population. Our analysis suggests that some variants (e.g., D38E) might not affect binding propensity while the potential impact of others (e.g., S19P) could not be determined. Further investigations on the effects of these rare variants on ACE2/SARS-CoV-2 binding affinity are needed.

When exploring patterns of codon evolution in ACE2, we found that multiple ACE2 residues important for the binding of SARS-CoV-2 S are evolving rapidly across mammals, with two (Q24 and H34) under positive selection (Fig. 4 *A* and *B* and *SI Appendix*, Fig. S5). Relative to other lineages analyzed, Chiroptera has a greater proportion of accelerated versus conserved codons (*SI Appendix*, Fig. S6), particularly in the SARS-CoV-2 S-binding region, suggesting the possibility of selective forces on these codons in Chiroptera driven by their interactions with SARS-CoV-2-like viruses (Fig. 4 *C* and *D* and Dataset S4.13). Indeed, distinct signatures of positive selection found in bat ACE2 (41) and in the SARS-CoV-2 S protein (42) support the hypothesis that bats are evolving to tolerate SARS-CoV-2-like viruses (discussed further below).

### Relationship of the ACE2 Binding Score to Known Infectivity of SARS-CoV-2.

Data on susceptibility of nonhuman species to SARS-CoV-2 is still very limited (*SI Appendix*, Fig. S10) but mostly agree with our predictions of ACE2 binding propensity for SARS-CoV-2 S (Figs. 1 and 2 and Dataset S1). Five out of six species with demonstrated susceptibility to SARS-CoV-2 infection score very high [rhesus macaque (43) and cynomolgus macaque (44)] or medium [domestic cat (45, 46), tiger (8) and golden hamster (47)]. Both species susceptible to infection but asymptomatic scored low [dog (45, 48) and Egyptian rousette bat (49)], and the three species resistant to infection scored either low [pig (45, 49)] or very low [mallard and red junglefowl (45, 49)].

A discrepancy was observed for ferret, which had a low ACE2 binding score but is susceptible to infection (45, 49–51). Ferrets may be a special case because of their unique respiratory biology (52). Ferrets are highly susceptible to upper respiratory tract infections and serve as models of respiratory diseases. They are susceptible to many viral diseases, including influenza type A and type B, canine distemper, and SARS-CoV (53). It has been proposed that ACE2 receptor distribution does not match the tropism of SARS-CoV in ferrets, because in ferrets viruses may use LSECTin receptor(s) to enable or enhance infectivity (52, 54). This may also be true for SARS-CoV-2 because the virus can potentially be glycosylated at 22 N-linked sites (55). Several studies have demonstrated SARS-CoV-2 infection in ferrets through intranasal inoculation of high doses (>105 plaque-forming units) of tissue-cultured virus, followed by direct or indirect transmission to naïve ferrets (45, 49–51). However, experimental infection via direct inoculation of high concentrations of tissue-cultured virus does not necessarily indicate infectability under natural conditions, and clinical signs of infection differed among studies. These data indicate that experimentally inoculated ferrets may become infected by another mechanism, possibly via high expression levels of low-affinity ACE2 and/or their very efficient LSECTin system.

### Mammals with Predicted High Risk of SARS-CoV-2 Infection.

Of the 19 catarrhine primates analyzed, 18/19 scored very high for binding of their ACE2 to SARS-CoV-2 S and one scored high (the Angola colobus); the 18 species scoring very high had 25/25 binding residues identical to human ACE2, including rhesus macaques, which are known to be infected by SARS-CoV-2 and develop COVID-19-like clinical symptoms (3, 43). Our analysis predicts that all Old-World primates are susceptible to infection by SARS-CoV-2 via ACE2. Thus, many of the 21 primate species native to China could be a potential reservoir for SARS-CoV-2. The remaining primate species were scored as high or medium, with only the gray mouse lemur and the Philippine tarsier scoring as low.

Although inconsistent with the species phylogeny, and overall similarity to human ACE2, we found that all three species of cervid deer and 12/14 cetacean species have high scores for binding of their ACE2s to SARS-CoV-2 S. There are 18 species of cervids found in China. While coronavirus sequences have been found in white-tailed deer (56) and gammacoronaviruses have been found in beluga whales (57, 58) and bottlenose dolphins (59), in which they are associated with respiratory diseases, the cellular receptor used by these viruses is not known. Studies of cellular infectivity in these species would provide important data for validating the prediction model.

### Other Artiodactyls.

A relatively large fraction (21/30) of artiodactyl mammals were classified with medium score for ACE2 binding to SARS-CoV-2 S. These include many species that are found in Hubei Province and around the world, such as domesticated cattle, sheep, and goats, as well as many species commonly found in zoos and wildlife parks (e.g., Masai giraffe, okapi, hippopotamus, water buffalo, scimitar-horned oryx, and dama gazelle). Although the cattle-derived MDBK cell line was shown in one study to be resistant to SARS-CoV-2 in vitro (60), our predictions suggest that ruminant artiodactyls can serve as a reservoir for SARS-CoV-2, which would have significant epidemiological implications as well as implications for food production and wildlife management (discussed below). It is noteworthy that camels and pigs, known for their ability to be infected by other coronaviruses (28), both score low in our analysis. These data are consistent with results (discussed above) indicating that pigs cannot be infected with SARS-CoV-2 either in vivo (45) or in vitro (60) but inconsistent with transfection studies using pig ACE2 receptors expressed in HeLa cells (1).

### Rodents.

Among the rodents, 7/46 species score high for ACE2 binding to SARS-CoV-2 S, and the remaining 11, 10, and 18 score medium, low, or very low, respectively. House mouse scored very low, consistent with infectivity studies (1, 60). Given that wild rodent species likely come in contact with bats as well as with other predicted high-risk species, rodents with high and medium scores cannot be excluded as possible intermediate hosts for SARS-CoV-2.

### Bats and Other Species of Interest.

Chiroptera represents a clade of mammals that are of high interest in COVID-19 research because several bat species are known to harbor coronaviruses, including those most closely related to SARS-CoV-2 (1). We analyzed ACE2 from 37 bat species, of which 8 and 29 scored low and very low, respectively. These results were intriguing because the three *Rhinolophus* spp. tested, including the Chinese rufous horseshoe bat, are major suspects in the transmission of SARS-CoV-2, or a closely related virus, to humans (1). Bats have been shown to harbor the highest diversity of betacoronaviruses among mammals (28) and show little pathology in individuals carrying these viruses (61).

Do bat ACE2 receptors bind SARS-CoV-2 S? Zhou et al. (1) transfected human ACE2-negative HeLa cells with ACE2 from a Chinese rufous horseshoe bat and obtained a low-efficiency infection with SARS-CoV-2. A recent report indicates that SARS-CoV-2 S protein can bind vesicular stomatitis virus (VSV) pseudotypes expressing halcyon horseshoe bat (*Rhinolophus alcyone*) ACE2 in BHK-21 cells (60). However, cell lines derived from big brown bat (*Eptesicus fuscus*) (62), Lander’s horseshoe bat (*Rhinolophus landeri*), and Daubenton’s bat (*Myotis daubentonii*) could not be infected with SARS-CoV-2 (60). Relatedly, cell lines from six different species of bats could not be infected with SARS-CoV, which also uses human ACE2 as a receptor (63). These data suggest that some bat species have evolved ACE2 receptors that do not bind SARS-CoV-like viruses or bind them with very low affinity, which is supported by our results showing positive selection and accelerated evolution of ACE2 in chiropterans. Alternatively, ACE2 expression could be very low in the bat cell lines, or SARS-CoV-2-like viruses can use other receptors, such as the MERS-CoV, a betacoronavirus that uses CD26/DPP4 (15), and porcine transmissible enteritis virus, an alphacoronavirus that uses aminopeptidase N (64). Also, other molecules required for SARS-CoV infection, such as TMPRSS2, might not be sufficiently expressed or function differently in bats.

Whether an ancestor of SARS-CoV-2, such as RaTG13, utilizes bat ACE2 is an important question related to whether bat ACE2 receptors bind SARS-CoV-2 S (discussed above). RaTG13 was found in feces of the intermediate horseshoe bat (*Rhinolophus affinis*) (1), but to our knowledge this virus has not been shown to bind to ACE2 of *R. affinis* or any other bat species. In addition, RaTG13 was reported not to infect human cells expressing *Rhinolophus sinicus* ACE2 in a recent study (65). Relatedly, Hoffman et al. (63) were unable to infect bat kidney- and lung-derived cell lines derived from six different species with VSV pseudotypes bearing SARS-CoV S protein or pseudotypes of two bat SARS-related CoV (Bg08 and Rp3) (63). Lack of concordance between the presence of bat SARS-CoV-like coronaviruses and binding to bat ACE2 may arise because of variations in susceptibility among bat species to SARS-CoV-like coronaviruses or due to one of the mechanisms discussed above.

### Carnivores.

Recent reports of a Malayan tiger and a domestic cat infected by SARS-CoV-2 suggest that the virus can be transmitted to other felids (8, 45). Our results are consistent with these studies; 9/9 felids we analyzed scored medium for ACE2 binding of SARS-CoV-2 S. However, the masked palm civet, a member of the Viverridae family that is related to but distinct from Felidae and proposed as the intermediate host for SARS-CoV, scored as very low. While our results are inconsistent with transfection studies using civet ACE2 receptors expressed in HeLa cells (1), these experiments have limitations as discussed above, and no data are available on infectivity in civet cells or animals. While carnivores closely related to dogs (dingoes, maned wolves, and foxes) all scored low, experimental data consistently show that dogs are not readily infected or symptomatic (45, 60, 66).

### Pangolins.

Considerable controversy surrounds reports that pangolins can serve as an intermediate host for SARS-CoV-2, with some reports proposing that SARS-CoV-2 arose as a recombinant between bat and pangolin betacoronaviruses (21, 67), while another study rejected that claim (68). In our study, ACE2 of Chinese pangolin, Sunda pangolin, and white-bellied pangolin had low or very low binding score for SARS-CoV-2 S. Binding of pangolin ACE2 to SARS-CoV-2 S was predicted using molecular binding simulations (67); however, neither experimental infection nor in vitro infection with SARS-CoV-2 has been reported for pangolins. Further studies are necessary to resolve whether SARS-CoV2 S binds to pangolin ACE2.

### Other Vertebrates.

Our analysis of species in 29 orders of fishes, 29 orders of birds, 3 orders of reptiles, and 2 orders of amphibians predicts that the ACE2 proteins of species within these vertebrate classes are not likely to bind SARS-CoV-2 S. Thus, vertebrate classes other than mammals are not likely to be an intermediate host or reservoir for the virus, despite predictions reported in a recent study (39), unless SARS-CoV-2 uses another receptor for infection. With diverse nonmammal vertebrates sold in the seafood and wildlife markets of Asia and elsewhere, it is important to determine if SARS-CoV-2 can be found in nonmammalian vertebrates.

### Animal Models for COVID-19.

Presently, there is a tremendous need for animal models to study SARS-CoV-2 infection and pathogenesis, as the only species currently known to be infected and show similar symptoms of COVID-19 is rhesus macaque. Nonhuman primate models have proven to be highly valuable for other infectious diseases but are expensive to maintain and numbers of experimental animals are limited. Our results provide an extended list of potential animal models for SARS-CoV-2 infection and pathogenesis, including large animals maintained for biomedical and agricultural research (e.g., domesticated sheep and cattle), and Chinese hamster and Syrian/golden hamster (47), which may be preferred due to their easier handling and already established value as models for other human diseases caused by viruses (69).

### Relevance to Threatened Species.

Among the 103 species that scored very high, high, and medium for ACE2/SARS-CoV-2 S binding, 41 (40%) are classified in one of three “threatened” categories (vulnerable, endangered, and critically endangered) on the International Union of Conservation of Nature (IUCN) Red List of Threatened Species, five are classified as near threatened, and two species are classified as extinct in the wild (70) (Dataset S1). This represents only a small fraction of the threatened species potentially susceptible to SARS-CoV-2. For example, all 20 catarrhine primate species in our analysis, representing three families (Cercopithecidae, Hylobatidae, and Hominidae) scored very high, suggesting that all 185 species of catarrhine primates, including 62 classified as threatened, are potentially susceptible to SARS-CoV-2. Similarly, all three species of deer, representatives of a family of ∼92 species (Cervidae), including 25 classified as threatened, scored as high. In contrast, some threatened species scored low or very low, such as the giant panda (low), potentially positive news for these at-risk populations.

In Cetacea, 12 of 14 species score as high, and of those two are threatened. Toothed whales have potential for viral outbreaks and have lost function of a gene that is key to the antiviral response in other mammalian lineages (71). If they are susceptible to SARS-CoV-2, human-to-animal transmission could pose a risk through sewage outfall (72) and contaminated refuse from cities, commercial vessels, and cruise liners (73). Our results have practical implications for populations of threatened species in the wild and those under human care (including those in zoos). Established guidelines for minimizing potential human-to-animal transmission should be implemented and strictly followed. Guidelines for field researchers working on great apes established by the IUCN have been in place since 2015 in response to previous human disease outbreaks (74) and have received renewed attention because of SARS-CoV-2 (74–76). For zoos, guidelines in response to SARS-CoV-2 have been distributed by several taxon advisory groups of the North American Association of Zoos and Aquariums, the American Association of Zoo Veterinarians, and the European Association of Zoo and Wildlife Veterinarians, and these organizations are actively monitoring and updating knowledge of species in human care considered to be potentially sensitive to infection (77, 78). Although in silico studies suggest potential susceptibility of diverse species, verification of infection potential is warranted, using cell cultures, stem cells, organoids, and other methods that do not require direct animal infection studies. Zoos and other facilities that maintain living animal collections are in a position to provide such samples for generating crucial research resources by banking tissues and cryobanking viable cell cultures in support of these efforts.

## Materials and Methods

### ACE2 Coding and Protein Sequences.

All human ACE2 orthologs for vertebrate species, and their respective coding sequences, were retrieved from NCBI Protein (20 March 2020) (79). ACE2 coding DNA sequences were extracted from available or recently sequenced genome assemblies for 123 other mammalian species, with the help of genome alignments and the human or within-family ACE2 orthologs. The protein sequences were predicted using AUGUSTUS v3.3.2 (80) or CESAR v2.0 (81) and the translated protein sequences were checked against the human ACE2 ortholog. ACE2 gene predictions were inspected and manually curated if necessary. For four bat species (*Micronycteris hirsuta*, *Mormoops blainvillei*, *Tadarida brasiliensis*, and *Pteronotus parnellii*) the *ACE2* coding region was split into two scaffolds which were merged, and for *Eonycteris spelaea* a putative 1-bp frameshift base error was corrected. Eighty ACE2 protein sequence predictions were obtained from the Zoonomia project, 19 from the Hiller Lab, 12 from the Koepfli laboratory, 8 from the Lewin laboratory, and 4 from the Zhao laboratory. The sources and accession numbers for the genomes or proteins retrieved from NCBI are listed in Dataset S1. The final set of ACE2 coding and protein sequences originated from 410 vertebrate species. To ensure alignment robustness, the full set of coding and protein sequences were aligned independently using Clustal Omega (82), MUSCLE (83), and COBALT (84), all with default parameters. All resulting protein alignments were identical. Clustal Omega alignments were used in the subsequent analysis. The classification of amino acid substitutions as conservative, semiconservative, and nonconservative were based on Clustal Omega definitions, which rely on the Gonnet Pam250 matrix scores. Briefly, a conservative substitution indicates a change to an amino acid with strongly similar biochemical/physicochemical properties, a semiconservative substitution depicts a change to an amino acid with weakly similar properties, and a nonconservative substitution depicts a change to an amino acid with no biochemical/physicochemical similarities.

### Identification of ACE2 Residues Involved in Binding to SARS-CoV-2 S Protein.

We identified 22 ACE2 protein residues that were previously reported to be critical for the effective binding of ACE2 RBD and SARS-CoV-2 S (12, 20). These residues include S19, Q24, T27, F28, D30, K31, H34, E35, E37, D38, Y41, Q42, L45, L79, M82, Y83, N330, K353, G354, D355, R357, and R393. All these residues were identified from the cocrystallization and structural determination of SARS-CoV-2 S and ACE2 RBD (12, 20). The known human ACE2 RBD glycosylation sites N53, N90, and N322 were also included in the analyzed residue set (10).

### ACE2 and SARS-CoV-2 Binding Ability Prediction.

Based on the known interactions of ACE2 and SARS-CoV-2 residues, we developed a set of rules for predicting the likelihood of the SARS-CoV-2 S binding to ACE2. These rules are primarily based on sequence similarity to the human ACE2 binding residues, with targeted rules applied to positions K353, K31, E35, M82, N53, N90, and N322 based on the effects of amino acid substitution on binding of SARS-CoV S (19). Sites N53, N90, and N322 are glycosylation sites at which disruption has been shown to affect viral attachment (10, 19). K353 and K31 are virus-binding hot spots; K353 establishes a salt bridge with ACE2 D38, and K31 forms a hydrogen bond with SARS-CoV-2 Q493 (12, 20). E35 supports the K31 binding hot spot by also establishing a hydrogen bond with SARS-CoV-2 Q493. The disruption of interactions at these residues, as well as the replacement of M82, were shown to significantly affect the attachment of SARS-CoV (19). Each species was classified in one of five categories: very high, high, medium, low, or very low potential for ACE2 binding to SARS-CoV-2 S. Species in the very high category have at least 23/25 critical residues identical to the human; have K353, K31, E35, M82, N53, N90, and N322; and have only conservative amino acid substitutions among the nonidentical 2/25 residues. Species in the high group have at least 20/25 residues identical to the human; have K353; have only conservative substitutions at K31 and E35; and can only have one nonconservative amino acid substitution among the 5/25 nonidentical residues. Species scoring medium have at least 20/25 residues identical to the human; can only have conservative substitutions at K353, K31, and E35; and can have up to two nonconservative amino acid substitutions in the 5/25 nonidentical residues. Species in the low category have at least 18/25 residues identical to the human; can only have conservative substitutions at K353; and can have up to three nonconservative amino acid substitutions on the remaining 7/25 nonidentical residues. Finally, species in the very low group have fewer than 18/25 residues identical to the human or have at least four nonconservative amino acid substitutions in the nonidentical residues.

### Protein Structure Analysis.

For 28 representative species, we modeled each exhibited individual variant onto the human structure 6MOJ (12), in the program CHIMERA (85), by choosing the rotamer with the least number of clashes, retaining the most initial hydrogen bonds, and containing the highest probability of formation as calculated by the CHIMERA program from the Dunbrack 2010 backbone-dependent rotamer library (*SI Appendix*, Fig. S9) (86). The chosen rotamer of the variant amino acid was then evaluated in the context of its structural environment and assigned a score based on the likelihood of interface disruption. “Neutral” was assigned if the residue maintained a similar environment as the original residue and was predicted to maintain or in some cases increase affinity. “Weakened” was assigned if hydrophobic contacts were lost and contacts that appear disruptive are introduced that are not technically clashes. “Unfavorable” was assigned if clashes are introduced and/or a hydrogen bond is broken. Potential for gross conformational changes between ACE2 proteins was checked by individually extracting a representative subset of the 28 species’ ACE2 proteins from the multiway alignment, which was then individually loaded into SWISS-Model (87) to generate homology-derived models. The output files were aligned to the template structure 6M18 (88), which is a cryo-electron microscopy model of the SARS-CoV-2 model. Because the amino acid sequences for the 28 species contained the transmembrane domain, the template 6M18 had the closest similarity relative to ACE2 crystal structures, which only contain the ectodomain. The quality of the models was assessed in SWISS-Model for coverage, sequence identity and global model quality estimation. The models were then imported to CHIMERA and the rmsd was calculated between the template structure and each individual model. Additional structural visualizations were generated in Pymol (89).

### Human Variants Analysis.

All variants at the 25 residues critical for effective ACE2 binding to SARS-CoV-2-S (10, 12, 20) were compiled from dbSNP (90), 1KGP (91), Topmed (92), UK10K (93), and CHINAMAP (24). Specific population frequencies were obtained from gnomAD v.2.1.1 (16).

### Phylogenetic Reconstruction of the Vertebrate *ACE2* Species Tree.

The multiple sequence alignment of 410 ACE2 orthologous protein sequences from mammals, birds, fishes, reptiles, and amphibians was used to generate a gene tree using the maximum likelihood method of reconstruction, as implemented in IQTREE (94). The best-fit model of sequence evolution was determined using ModelFinder (95) and used to generate the species phylogeny. A total of 1,000 bootstrap replicates were used to determine node support using UFBoot (96).

### Identifying Sites Undergoing Positive Selection.

Signatures of site-specific positive selection in the ACE2 receptor were explored using CodeML, part of the Phylogenetic Analysis using Maximum Likelihood (PAML) (97) suite of software. Given CodeML’s computational complexity, a smaller subset of mammalian taxa (*n* = 64; Dataset S1), which included species from all prediction categories mentioned above, was used for selection analyses. To calculate likelihood-derived dN/dS rates (⍵), CodeML utilizes both a species tree and a codon alignment. The species tree for all 64 taxa was calculated using IQTREE (94) and the inferred best-fit model of sequence evolution (JTT+F + R4). This gene topology was generally in agreement with the 410 taxa tree; however, bats were now sister taxa to Perissodactyla. Therefore, all selection analyses were run using both the inferred gene tree and a modified tree with the position of bats manually modified to reflect the 410 taxa topology. All species trees used were unrooted. A codon alignment of the 64 mammals was generated using pal2nal (98) with protein alignments generated with Clustal Omega (82) and their respective coding sequences.

Site models M7 (null model) and M8 (alternative model) were used to identify *ACE2* sites undergoing positive selection in mammals. Both M7 and M8 estimate ⍵ using a beta distribution and 10 rate categories per site with ⍵ ≤ 1 (neutral or purifying selection) but with an additional 11th category allowing ⍵ >1 (positive selection) in M8. An LRT calculated as 2*(lnL_alt_ – lnL_null_), comparing the fit of both null and alternative model likelihoods was carried out, with a *P* value calculated assuming a χ^2^ distribution. Sites showing evidence of positive selection were identified by a significant (>0.95) BEB score and validated by visual inspection of the protein alignment. To explore order-specific instances of positive selection, separate multiple sequence alignments and gene trees for Chiroptera (*n* = 37), Cetartiodactyla (*n* = 45), Carnivora (*n* = 44), Rodentia (*n* = 46), and Primates (*n* = 39) were also generated and explored using M7 vs. M8 in CodeML. The M0 model in CodeML was used to explore consistency across parameters inferred maximum likelihood (e.g., transition/transversion rates and branch lengths).

In addition to site models, branch-site model A1 (null model) and model A (alternative model) were also implemented targeting various mammalian orders, specifically Chiroptera, Cetartiodactyla, Rodentia, and Primates, to identify lineage-specific positive selection in the *ACE2* receptor sequence. Branch-site Model A1 constrains both the target foreground branch (Carnivora, Chiroptera, Cetartiodactyla, Rodentia, and Primates) and background branches to ⍵ ≤ 1, while the alternative Model A allows positive selection to occur in the foreground branch. Null and alternative models were compared using LRTs as above, with significant BEB sites identified.

We also looked for positively selected sites in the viral spike protein, using coding sequences from 43 SARS-CoV-2, SARS-CoV, and CoV-like viral strains. Protein and codon alignments were generated as above, with the viral species tree inferred using the spike alignment generated with Clustal Omega. Site-test models were applied using CodeML and significant BEB sites identified.

### Analysis for Departure from Neutral Evolutionary Rate in ACE2 with PHAST.

Neutral models were trained on the specified species sets (Dataset S4) using the REV nucleotide substitution model implemented in phyloFit using an expectation-maximization algorithm for parameter optimization. The neutral model fit was based on third-codon positions to approximate the neutral evolution rate specific to the *ACE2* gene, using a 410-species phylogenetic tree generated by IQTREE as described above and rooted on fishes. The program phyloP was then used to identify codons undergoing accelerated or conserved evolution relative to the neutral model using –features to specify codons, –method LRT –mode CONACC, and –subtree for lineage-specific tests, with *P* values thus assigned per codon based on an LRT. *P* values were corrected for multiple testing using the Benjamini–Hochberg method (99) and sites with a corrected *P* value less than 0.05 were considered significant. PhyloFit and phyloP are both part of the PHAST package v1.4 (100, 101). In order to assess the relative power among the various clades, we followed a simulation-based protocol (99). Using the program phyloBoot from PHAST, we generated 1,000 alignments of length 2,415 nucleotides to match the size of the *ACE2* codon alignment for different subtree scaling factors (e.g., phyloBoot -L 2415 -n 1000 -t tree.nh -l 1.11 -S Chiroptera mammals.CDS-3.mod -a out_root) (100, 101). Lambda represents the scale of the departure from neutral evolution in a clade, with lambda less than one indicating conservation and greater than one indicating acceleration. Greater values of lambda indicate greater amounts of acceleration or effect size and thus require less power to detect. We then ran phyloP on these alignments with the same parameters as used to test the ACE2 alignment for each clade and determined the number of accelerated codons at each value of lambda for each clade (*SI Appendix*, Fig. S8). The simulator generates nucleotide (not amino acid) sequences and is therefore conservative in its estimations of power for acceleration but adequate for defining relative power between clades. These results are concordant with the summed branch lengths identified using tree_doctor from PHAST (100, 101) for each clade (*SI Appendix*, Table S2), which is expected as previous analyses found power to detect departures from neutral evolution to scale with subtree length (99).

## Supplementary Material

Supplementary File

Supplementary File

Supplementary File

Supplementary File

Supplementary File

## Data Availability

All accession numbers or genome availability for the 410 species used in this study are listed in Dataset S1. This study made use of ACE2 protein sequences previously available from NCBI protein database (*n* = 287) and ACE2 sequences extracted from genomes previously available from NCBI assembly (*n* = 106) (102). ACE2 sequences were extracted from the genomes of Bowhead whale (available at http://alfred.liv.ac.uk/downloads/bowhead_whale/bowhead_whale_scaffolds.zip), velvety free-tailed bat (available at https://vgp.github.io/genomeark/Molossus_molossus/), greater mouse-eared bat (available at https://vgp.github.io/genomeark/Myotis_myotis/), Kuhl’s pipistrelle (available at https://vgp.github.io/genomeark/Pipistrellus_kuhlii/), scimitar oryx (available at https://www.dnazoo.org/assemblies/Oryx_dammah), and white-bellied pangolin (available at https://www.dnazoo.org/assemblies/Phataginus_tricuspis). The ACE2 sequences of Pratt’s roundleaf bat, Pearson’s horseshoe bat, greater short-nosed fruit bat, and Indian false vampire were submitted to NCBI under the accession nos. MT515621–MT515624. The ACE2 sequences of dama gazelle, Sunda clouded leopard, clouded leopard, maned wolf, bush dog, European mink, and black-footed ferret were also submitted to NCBI and are available under the accession nos. MT560518–MT560524.
