# Supplementary material for: Broad host range of SARS-CoV-2 predicted by comparative and structural analysis of ACE2 in vertebrates
Source: Proc Natl Acad Sci U S A. 2020 Aug 21;117(36):22311–22. doi: 10.1073/pnas.2010146117 (PMC7486773; doi:10.1073/pnas.2010146117)
Supplement: Supplementary File [file pnas.2010146117.sapp.pdf]

Supplementary Information for

Broad Host Range of SARS-CoV-2 Predicted by Comparative and Structural Analysis of ACE2 in Vertebrates

Joana Damas, Graham M. Hughes, Kathleen C. Keough, Corrie A. Painter, Nicole S. Persky, Marco Corbo, Michael Hiller, Klaus-Peter Koepfli, Andreas R. Pfenning, Huabin Zhao, Diane P. Genereux, Ross Swofford, Katherine S. Pollard, Oliver A. Ryder, Martin T. Nweeia, Kerstin Lindblad-Toh, Emma. C. Teeling, Elinor K. Karlsson, and Harris A. Lewin

Corresponding author: Harris A. Lewin, lewin@ucdavis.edu

This PDF file includes:

Supplementary text
Figures S1 to S8
Legends for Datasets S1 to S4
SI References

Other supplementary materials for this manuscript include the following:

Datasets S1 to S4

www.pnas.org/cgi/doi/10.1073/pnas.2010146117

Supplementary Information Text

Comparison of protein structure analysis methodologies. Of the 16 residues in the structural homology assessment performed in this study (Fig. S1), just one *unfavorable* substitution, K353H, differentiated mouse from the four other species tested in the Zhou and collaborators study (human, pig, Masked palm civet and Chinese rufous horseshoe bat)(1). Mouse was the single species whose ACE2 did not bind SARS-CoV-2 in that study (1). The K353H substitution has been shown experimentally to abrogate binding of ACE2 to SARS-CoV spike protein (2). We used this site to test another recently published approach that assessed binding affinity between ACE2 and SARS-CoV-2 using theoretical changes in Gibb's free energy (3). We estimated the change in Gibbs free energy for the K353H substitution for both SARS-CoV, using the template PDB 2AJF as well as the SARS-CoV-2 using the PDB 6M0J using the SSIPe program (4). The output suggested that the $\Delta\Delta G_{bind}$ in the SARS-CoV-2 would be 0.460 kcal/mol and in the SARs-CoV 0.594 kcal/mol, neither of which would be predicted to cause a disruption in affinity significant enough to abrogate binding (3, 4). Therefore, despite offering the potential to quantify predicted binding affinities, without information from experimentally derived binding assays, the Gibb's free energy method may not accurately represent the impact of different substitutions along the binding interface.

Another recent body of work emerged while this manuscript was under preparation, which provided a wealth of experimentally derived insights into the impact on binding to SARS-CoV-2 Spike protein when each residue of the ACE2 binding interface was substituted for each amino acid (5). We compared the predictions that we generated for residues that varied along the binding interface from 28 species with the results obtained by Procko, to determine the percent discordance and concordance between approaches (Table S3). Discordant values were calculated as 16.6% of the 55 substitutions analyzed using the following rules: discordant substitutions are designated as *neutral* (N) from this analysis and (--) from the Procko analysis; *unfavorable* (U) from this analysis and (0, +, ++) from the Procko analysis; *weaken* (W) from this analysis and (+, ++) from the Procko analysis according to Table S3. Residues that were calculated to be discordant between these two analyses were indicated by an (*) in Fig. S1.

	Species		Q24	T27	D30	K31	H34	E35	E37	D38	Y41	Q42	L45	L79	M82	Y83	K353	G354
High	Odocoileus virginianus texanus	*White-tailed deer*	. .	. .	E N	. .	. .	. .	. .	. .	. .	. .	. .	M N	T N	. .	. .	. .
	Rangifer tarandus	*Reindeer*	. .	. .	E N	. .	. .	. .	. .	. .	. .	. .	. .	M N	T N	. .	. .	. .
	Eulemur flavifrons	*Blue-eyed black lemur*	E N	A N	. .	. .	. .	. .	. .	. .	. .	. .	. .	. .	T N	. .	. .	. .
	Propithecus coquereli	*Coquerel's sifaka*	. .	. .	. .	. .	. .	. .	. .	. .	. .	. .	. .	. .	T N	. .	. .	. .
	Dipodomys stephensi	*Stephens's kangaroo rat*	L U	. .	. .	N W	Q N	. .	. .	. .	. .	. .	. .	. .	I N	. .	. .	. .
Medium	Bos taurus	*Cattle*	. .	. .	E N	. .	. .	. .	. .	. .	. .	. .	. .	M N	T N	. .	. .	. .
	Felis catus	*Cat*	L U	. .	E N	. .	. .	. .	. .	E N	. .	. .	. .	. .	T N	. .	. .	. .
	Panthera tigris altaica	*Siberian tiger*	L U	. .	E N	. .	. .	. .	. .	E N	. .	. .	. .	. .	T N	. .	. .	. .
	Mesocricetus auratus	*Golden hamster*	. .	. .	. .	. .	Q N	. .	. .	. .	. .	. .	. .	. .	N U*	. .	. .	. .
Low	Sus scrofa	*Pig*	L U	. .	E N	. .	L U	. .	. .	. .	. .	. .	. .	I N	T N	. .	. .	. .
	Ailuropoda melanoleuca	*Giant panda*	L U	. .	E N	. .	Y U	. .	. .	. .	. .	. .	. .	H W	T N	. .	. .	. .
	Canis lupus familiaris	*Dog*	L U	. .	E N	. .	Y U	. .	. .	E N	. .	. .	. .	. .	T N	. .	. .	. .
	Rhinolophus pearsonii	*Pearson's horseshoe bat*	. .	I N	. .	. .	R N	. .	. .	. .	H W	E W	. .	. .	D U	. .	. .	D U
	Dipodomys ordii	*Ord's kangaroo rat*	L U	. .	. .	N W	Q N	. .	. .	. .	. .	. .	. .	. .	I N	. .	. .	. .
Very Low	Catagonus wagneri	*Chacoan peccary*	L U	. .	E N	. .	L U	. .	. .	. .	. .	. .	. .	T W*	T N	. .	. .	. .
	Mustela putorius furo	*Ferret*	L U	. .	E N	. .	Y U	. .	. .	E N	. .	. .	. .	H W	T N	. .	. .	R U
	Paguma larvata	*Masked palm civet*	L U	. .	E N	T U	Y U	. .	Q N	E N	. .	. .	V N	. .	T N	. .	. .	. .
	Hipposideros armiger	*Great roundleaf bat*	L U	E U	. .	. .	T U*	. .	. .	. .	H W	L U*	. .	R W*	D U	. .	. .	. .
	Hipposideros galeritus	*Cantor's roundleaf bat*	S U	I N	. .	. .	T U*	D W*	. .	E N	H W	. .	. .	. .	D U	. .	. .	. .
	Hipposideros pratti	*Pratt's roundleaf bat*	L U	E U	. .	. .	T U*	. .	. .	. .	H W	L U*	. .	R W*	D U	. .	. .	. .
	Rhinolophus ferrumequinum	*Greater horseshoe bat*	L U	K U*	. .	D W	S W*	. .	. .	N N	H W	. .	. .	. .	N U*	F W	. .	. .
	Uropsilus gracilis	*Gracile shrew mole*	. .	E U	E N	N W	R N	N W	. .	N N	. .	K N/A	. .	I N	Q U	F W	M W	K U
	Manis javanica	*Sunda pangolin*	E N	. .	E N	. .	S W*	. .	. .	E N	. .	. .	. .	I N	N U*	. .	. .	H U
	Manis pentadactyla	*Chinese pangolin*	E N	. .	E N	. .	S W*	. .	. .	E N	. .	. .	. .	I N	N U*	. .	. .	H U
	Otolemur garnettii	*Northern greater galago*	. .	. .	. .	N W	R N	. .	. .	E N	H W	. .	. .	I N	T N	. .	. .	D U
	Mus musculus	*House mouse*	N U	. .	N N	. .	Q N	. .	. .	. .	. .	. .	. .	T W*	S U	F W	H U	. .

* discordant with Procko et al

Fig. S1. Evaluation of binding contacts between host ACE2 and SARS-CoV-2 in 28 representative species selected from *very low*, *low*, *medium* and *high* binding score groups, and for each residue in the ACE2 binding interface that varied from human (55 substitutions in 16 residues). For each residue, amino acid substitutions are shown on the left as white boxes, with sites matching human ACE2 shown in gray. For each residue, the evaluation of the binding contact is shown on the right as *neutral* (N; blue box), w*eakening* (W; orange box); or *unfavorable* (U; red box), with sites matching human ACE2 in blue. Evaluations discordant with Procko (5) are marked with an asterisk and lighter background color.

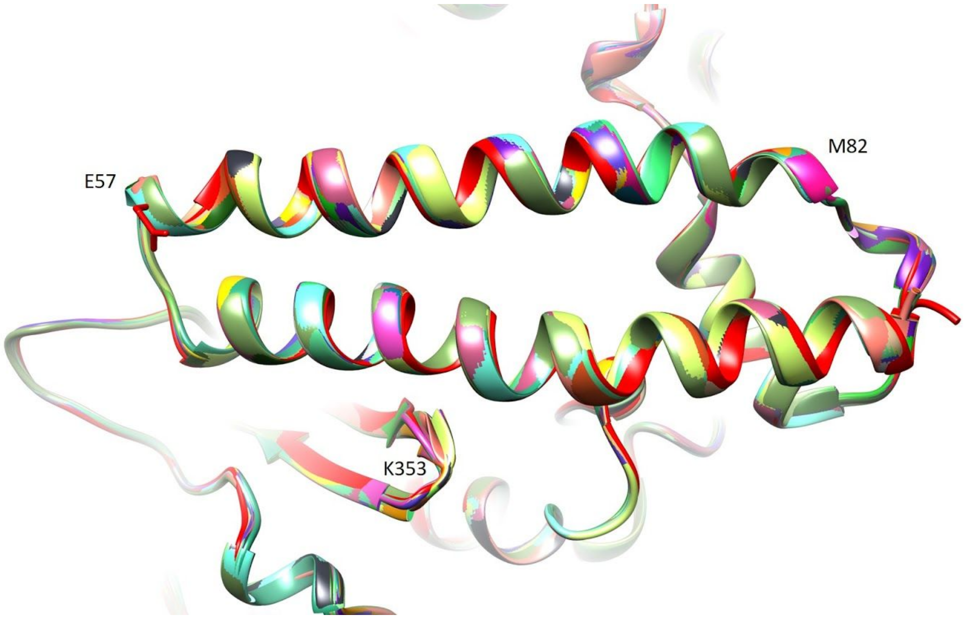

Fig. S2. Homology models generated from SWISS-Model were aligned in Chimera. The Human ACE2 template structure is shown in red cartoon with 28 additional species overlaid in order to show concordance with the template structure. Positions of two individual residues along the binding interface (M82, K353) and one outside (E57) are shown.

Fig. S3. Phylogenetic tree of ACE2 proteins in mammals, rooted on fish.

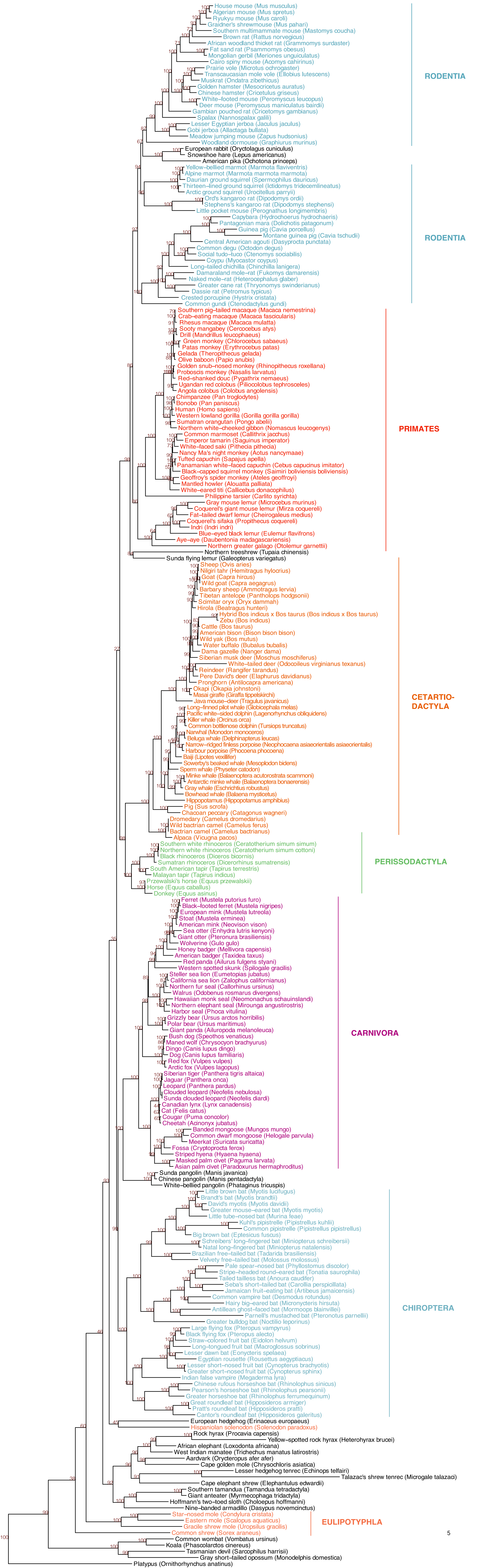

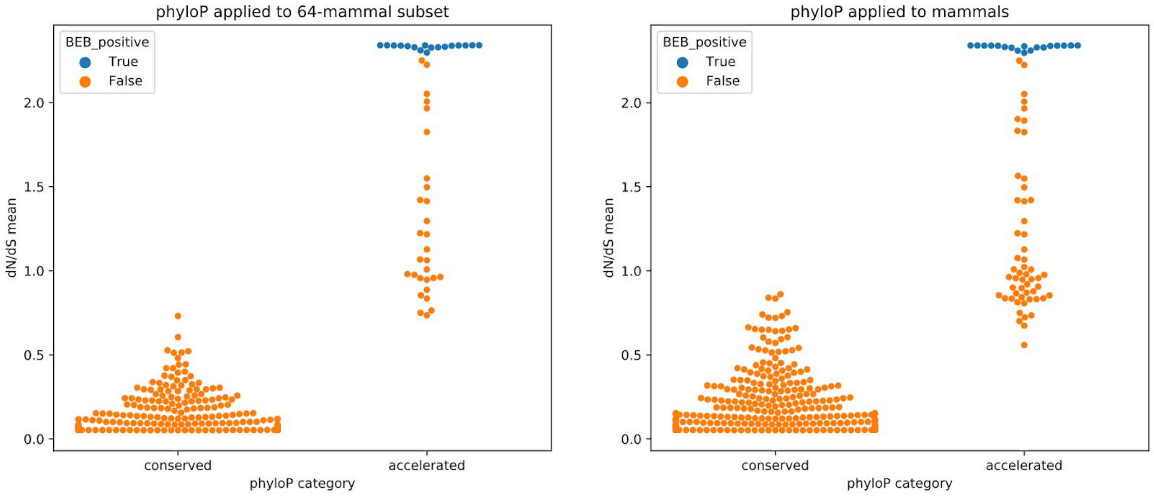

Fig. S4. Significant results from phyloP, both conserved and accelerated, for ACE2 codons compared with CodeML BEB scores. Left panel shows phyloP results for the 64-mammals subset used in the mammal CodeML analysis. Right panel shows phyloP results for all mammals in the alignment. The y-axis represents dN/dS values calculated by CodeML, x-axis indicates whether the codons were classified as conserved or accelerated by phyloP. All dots are significant results from phyloP, blue dots are also significantly positively selected from CodeML.

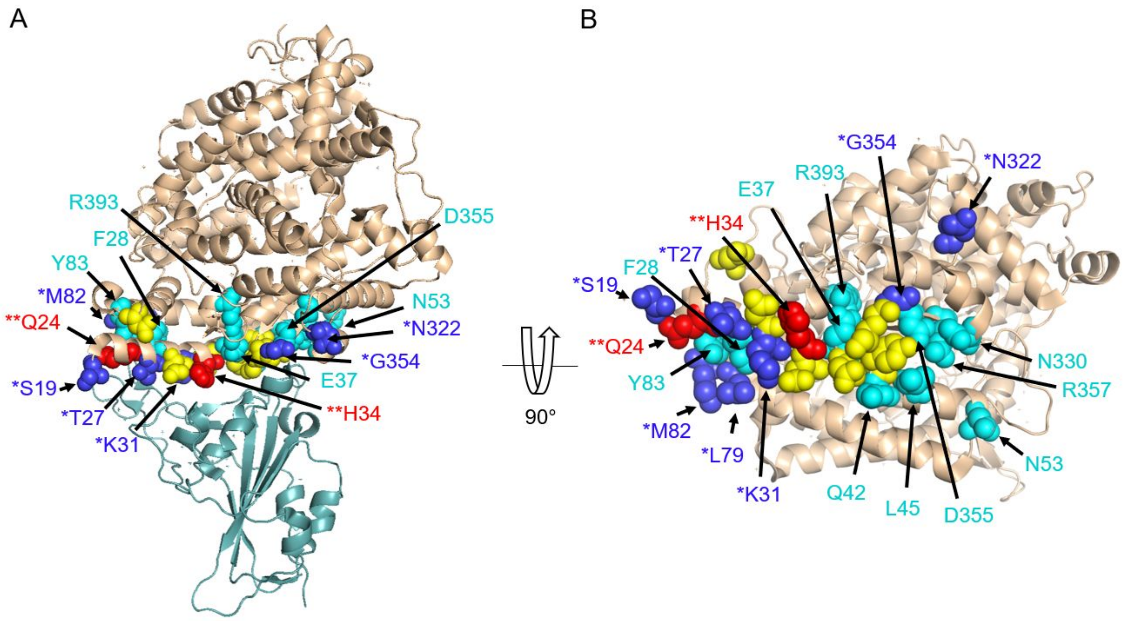

Fig. S5. Residues under accelerated evolution in mammals, overlapping the binding interface, as detected using phyloP. (**A**) The SARS-CoV-2 spike RBD is shown in light teal cartoon. ACE2 is shown in wheat cartoon with residues involved in the binding interface shown in yellow spheres. (*) Dark blue and red spheres indicate ACE2 residues that are accelerated, under positive selection and overlapping the binding interface. Cyan spheres indicate ACE2 residues that are conserved. (**) Red spheres also demonstrate positive selection with CodeML. (**B**) 90 degree rotation of the ACE2 protein.

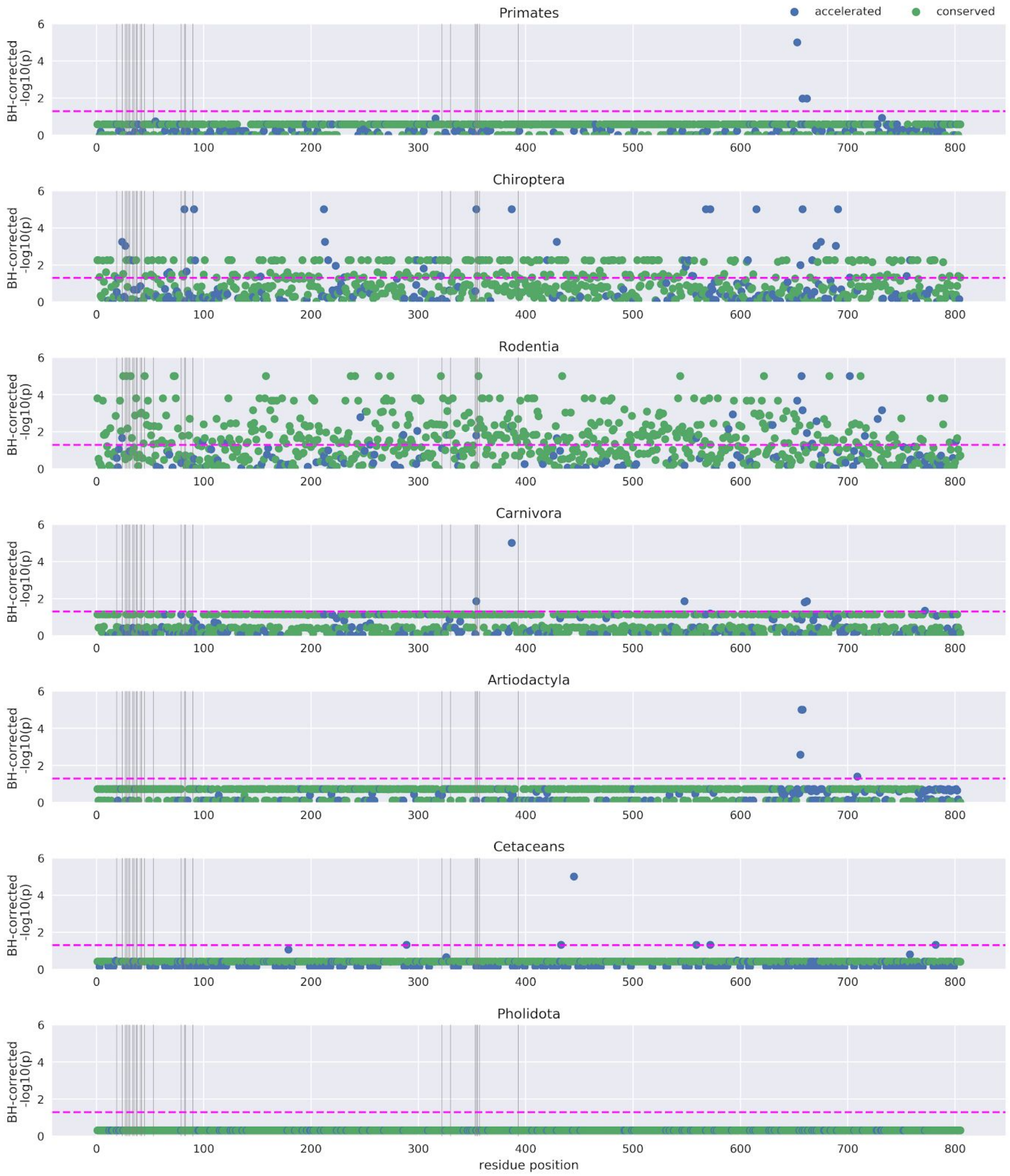

Fig. S6. Intralineage phyloP results for all ACE2 codons. PhyloP signal was assessed at all ACE2 codons for various mammalian lineages against neutral models trained on those lineages, thereby identifying intralineage signals of shifts in evolutionary rate. Green dots indicate codons classified as conserved and blue dots accelerated. Vertical grey lines indicate important binding residues in ACE2. The x-axis indicates the corresponding position in the ACE2 protein for each codon, and the y-axis indicates the phyloP p-value for each codon.

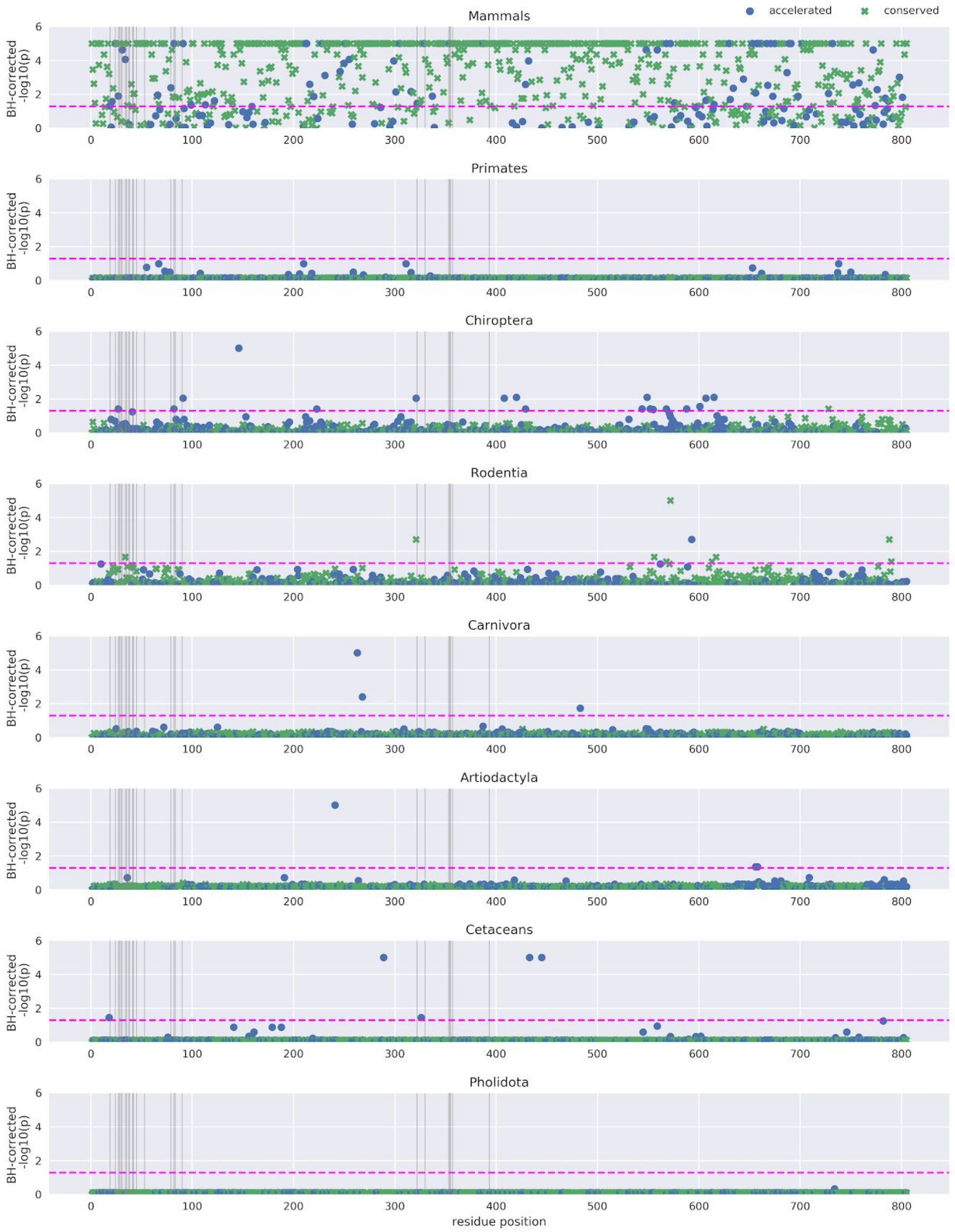

Fig. S7. PhyloP results for mammalian lineages against a mammal neutral model. PhyloP signal was assessed at all ACE2 codons for various mammalian lineages against a neutral model trained on all mammalian species in the alignment. Green dots indicate codons classified as conserved and blue dots accelerated. Vertical grey lines indicate important binding residues in ACE2. The x-axis indicates the corresponding position in the ACE2 protein for each codon, and the y-axis indicates the phyloP p-value for each codon.

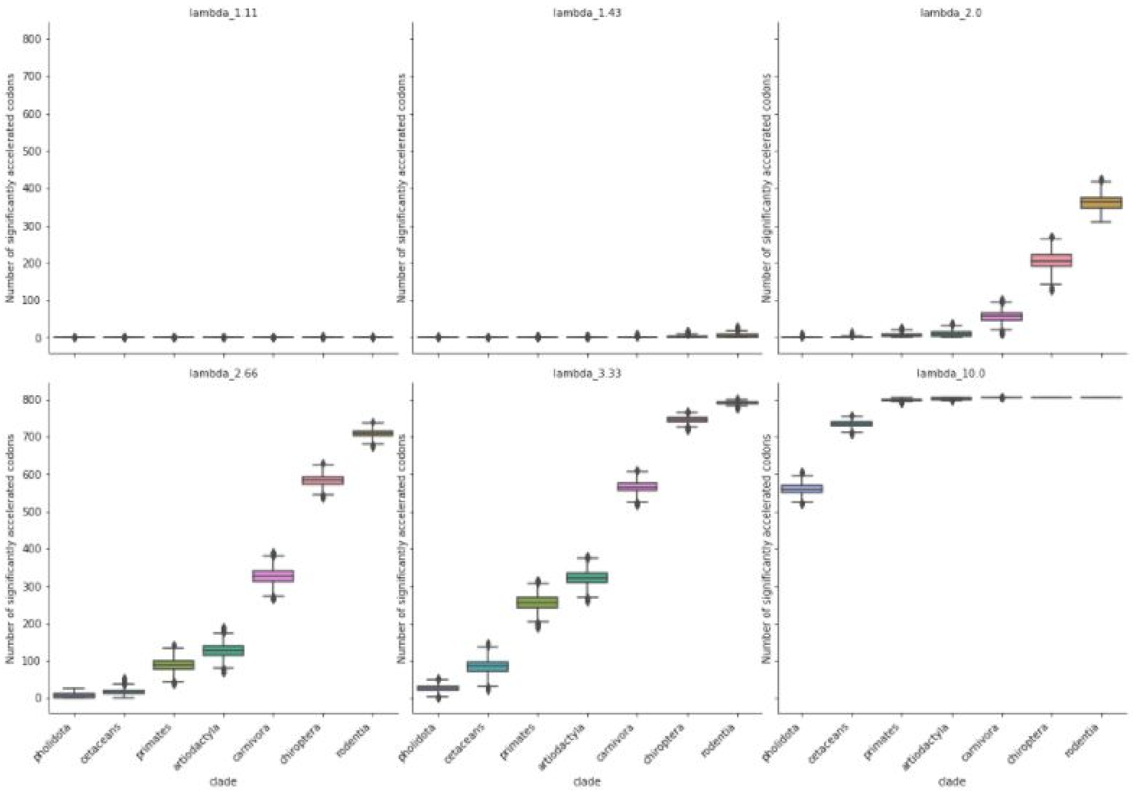

Fig. S8. Power to detect acceleration relative to mammals among the clades for various values of lambda. 1000 alignments of length 2415 nucleotides, the same length as *ACE2*, were generated at varying values of lambda and phyloP applied to determine the number of accelerated codons that could be identified. Lambda is the branch scaling factor for the clade subtree, greater values of lambda indicate greater acceleration within the clade. The greatest number of accelerated codons could be identified in Rodentia, indicating the greatest level of power for that clade.

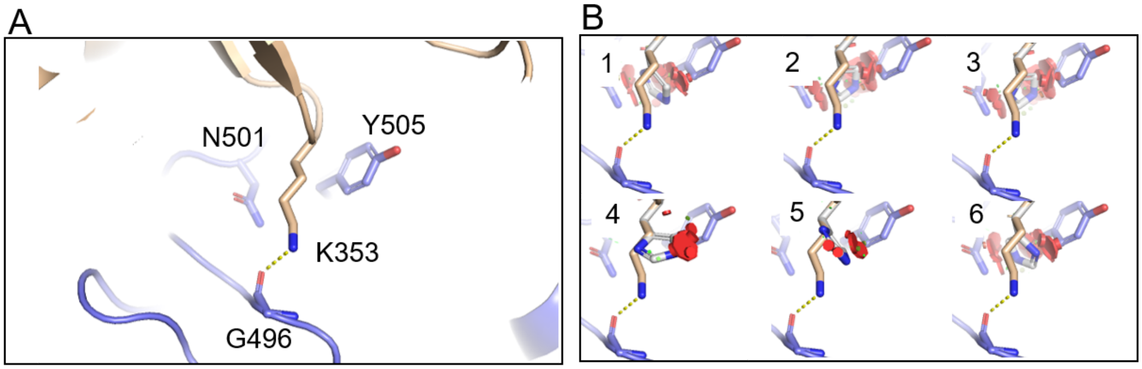

Fig. S9. Selecting the best-fit rotamer for K353H. (**A**) Cartoon representation of ACE2 K353 depicted in wheat stick with a dotted line representing the hydrogen bond to SARS-CoV-2 spike (S) G496. Y505 and N501 from the S RBD, which are contact residues for ACE2 K353 are shown. (**B**) All Dunbrack derived rotamers for histidine show clashes as red disks at this position, with rotamer 5 showing the least steric hindrance. In addition, substitution to histidine at this position would eliminate the hydrogen bond between the 353 position on ACE2 and the S RBD at G496. This substitution was designated as being potentially *unfavorable* due to the loss of the hydrogen bond in addition to the steric hindrance.

					Reported susceptibility to SARS-CoV-2			References		
Class	Order	Common name	Species	Binding score	Animals	Animal cell lines	Human cell line expressing species ACE2	Animal studies	Animal cell lines studies	Human cell line expressing species ACE2 studies
Mammalia	Primates	Rhesus macaque	*Macaca mulatta*	VERY HIGH	Infectable; severe disease	no data	no data	Munster 2020; Shan 2020		
Mammalia	Primates	Cynomolgus Macaque	*Macaca fascicularis*	VERY HIGH	Infectable; severe disease	no data	no data	Rockx 2020		
Mammalia	Primates	Green monkey	*Chlorocebus sabaeus*	VERY HIGH	no data	Yes	no data		Harcourt 2020; Wang 2020; Hoffman 2020	
Mammalia	Rodentia	Golden hamster	*Mesocricetus auratus*	MEDIUM	Infectable; severe disease	No	no data	Chan 2020	Hoffman 2020	
Mammalia	Carnivora	Cat	*Felis catus*	MEDIUM	Infectable; mild disease or asymptomatic	no data	no data	Shi 2020; Halfmann 2020		
Mammalia	Artiodactyla	Cattle	*Bos taurus*	MEDIUM	no data	No	no data		Hoffman 2020	
Mammalia	Carnivora	Dog	*Canis lupus familiaris*	LOW	Infectable but asymptomatic	Yes	no data	Shi 2020; Sit 2020	Hoffman 2020	
Mammalia	Chiroptera	Egyptian rousette	*Rousettus aegyptiacus*	LOW	Infectable but asymptomatic	no data	no data	Schlottau 2020		
Mammalia	Artiodactyla	Pig	*Sus scrofa*	LOW	No	Conflicting results	Yes	Shi 2020; Schlottau 2020	Hoffman 2020; Chu 2020	Zhou 2020
Mammalia	Chiroptera	Chinese rufous horseshoe bat	*Rhinolophus sinicus*	LOW	no data	No	Yes		Chu 2020	Zhou 2020
Mammalia	Carnivora	Ferret	*Mustela putorius furo*	VERY LOW	Infectable; mild disease or asymptomatic	no data	no data	Shi 2020; Schlottau 2020		
Mammalia	Rodentia	House mouse	*Mus musculus*	VERY LOW	no data	No	No		Hoffman 2020	Zhou 2020
Mammalia	Chiroptera	Big brown bat	*Eptesicus fuscus*	VERY LOW	no data	No	no data		Harcourt 2020	
Mammalia	Carnivora	Masked palm civet	*Paguma larvata*	VERY LOW	no data	no data	Yes			Zhou 2020
Aves	Anseriformes	Mallard	*Anas platyrhynchos*	VERY LOW	No	no data	no data	Shi 2020		
Aves	Galliformes	Red junglefowl	*Gallus gallus*	VERY LOW	No	no data	no data	Shi 2020; Schlottau 2020		
Mammalia	Chiroptera	Halcyon horseshoe bat	*Rhinolophus alcyone*	N/A	no data	No	Yes		Hoffman 2020	Hoffmann 2020
Mammalia	Chiroptera	Daubenton's bat	*Myotis daubentonii*	N/A	no data	No	no data		Hoffman 2020	

Fig. S10. Summary of available SARS-CoV-2 susceptibility data for vertebrate species. Data on susceptibility of non-human species to SARS-CoV-2 is still very limited, but information available to date suggests that species we rank as very high or medium risk are more likely to be infected and symptomatic than those ranked as low or very low. Susceptibility classifications are based on data published in Chan 2020 (6), Chu 2020 (7), Halfmann 2020 (8), Hoffmann 2020 (9), Rockx 2020 (10), Shi 2020 (11), Sit 2020 (12), Wang 2020 (13) and Zhou 2020 (1), and in preprints Harcourt 2020 (14), Munster 2020 (15), Schlottau 2020 (16) and Shan 2020 (17).

Table S1. Homology Model Assessment. Homology models derived from SWISS-Model for the ACE2 protein from 28 species were assessed for sequence identity and coverage, GMQE (Global Model Quality Estimation) and QMEAN (absolute quality estimates on the basis of one single model). Each model was also aligned with the template structure 6M18 to calculate the root mean square deviation (RMSD).

Species	Sequence identity (%)	Sequence coverage	Range (residues)	GMQE	QMEAN	RMSD
White-tailed deer	82.74	0.91	21-631	0.82	-0.86	0.06
Reindeer	82.09	0.99	21-767	0.86	-1.53	0.12
Blue-eyed black lemur	84.39	0.98	21-768	0.89	-1.06	0.14
Coquerel's sifaka	85.79	0.98	21-768	0.9	-1.2	0.09
Stephens's kangaroo rat	82.24	0.99	21-768	0.87	-1.16	0.12
Cattle	81.21	0.99	21-767	0.86	-0.98	0.07
Cat	85.39	0.99	21-768	0.88	-1.45	0.08
Siberian tiger	85.77	1	13-760	0.91	-1.39	0.08
Golden hamster	84.26	0.99	21-768	0.89	-1	0.07
Pig	81.08	0.99	21-768	0.86	-1.51	0.09
Giant panda	83.42	0.97	22-768	0.87	-1.55	0.09
Dog	83.55	0.98	21-767	0.86	-1.66	0.12
Pearson's horseshoe bat	81.61	0.99	21-768	0.85	-1.6	0.08
Ord's kangaroo rat	82.12	0.99	21-768	0.87	-1.13	0.12
Chacoan peccary	81.72	0.99	21-768	0.86	-1.47	0.09
Ferret	82.78	0.97	22-768	0.86	-1.44	0.13
Masked palm civet	83.63	0.99	21-768	0.87	-1.39	0.09
Great roundleaf bat	80.46	0.98	21-769	0.84	-1.5	0.1
Cantor's roundleaf bat	79.70	0.98	21-769	0.84	-1.42	0.13
Pratt's roundleaf bat	80.46	0.98	21-769	0.84	-1.53	0.1
Greater horseshoe bat	81.36	0.99	21-768	0.85	-1.4	0.17
Gracile shrew mole	74.97	0.98	21-763	0.82	-1.18	0.14
Sunda pangolin	84.76	0.99	21-768	0.87	-1.6	0.09
Chinese pangolin	85.14	0.99	21-768	0.87	-1.53	0.09
Northern greater galago	81.74	0.99	21-768	0.87	-1.22	0.07
House mouse	81.86	0.99	21-768	0.86	-1.28	0.01

Table S2. Summed branch length for each clade subtree.

**Clade**	**Summed Branch Length**
Pholidota	0.042032
Cetaceans	0.151972
Primates	0.235031
Artiodactyla	0.416438
Carnivora	0.574820
Chiroptera	1.574263
Rodentia	1.942557

Table S3. Comparing structure-based predictions and experimentally derived values for the impact of substitutions along the ACE2/SARS-CoV-2 spike RBD interface.

Current work	Procko	Number matched
N	-	13
N	+	1
N	++	3
N	0	1
U	1	12
U	--	7
U	++	2
U	0	2
W	-	8
W	+	4
W	++	1
W	0	1

Dataset S1 (separate file). Variation at 25 residues critical for ACE2 and SARS-CoV-2 binding in 410 vertebrate species.

Dataset S2 (separate file). Table showing the human variant analysis on the 25 critical ACE2 binding residues.

Dataset S3 (separate file). Phylogenetic tree of ACE2 proteins in 410 vertebrate species, rooted on fish. Bootstrap support values are displayed.

Dataset S4 (separate file). Table with results from conservation, acceleration and selection analyses with phyloP and CodeML.

SI References

1. P. Zhou, *et al.*, A pneumonia outbreak associated with a new coronavirus of probable bat origin. *Nature* **579**, 270–273 (2020).

2. W. Li, *et al.*, Receptor and viral determinants of SARS-coronavirus adaptation to human ACE2. *EMBO J.* **24**, 1634–1643 (2005).

3. A. D. Melin, M. C. Janiak, F. Marrone, P. S. Arora, J. P. Higham, Comparative ACE2 variation and primate COVID-19 risk. *bioRxiv*, 2020.04.09.034967 (2020).

4. X. Huang, W. Zheng, R. Pearce, Y. Zhang, SSIPe: accurately estimating protein-protein binding affinity change upon mutations using evolutionary profiles in combination with an optimized physical energy function. *Bioinformatics* (2019) https:/doi.org/10.1093/bioinformatics/btz926.

5. E. Procko, The sequence of human ACE2 is suboptimal for binding the S spike protein of SARS coronavirus 2. *bioRxiv*, 2020.03.16.994236 (2020).

6. J. F.-W. Chan, *et al.*, Simulation of the clinical and pathological manifestations of Coronavirus Disease 2019 (COVID-19) in golden Syrian hamster model: implications for disease pathogenesis and transmissibility. *Clin. Infect. Dis.* (2020) https:/doi.org/10.1093/cid/ciaa325.

7. H. Chu, *et al.*, Comparative tropism, replication kinetics, and cell damage profiling of SARS-CoV-2 and SARS-CoV with implications for clinical manifestations, transmissibility, and laboratory studies of COVID-19: an observational study. *The Lancet Microbe* **1**, e14–e23 (2020).

8. P. J. Halfmann, *et al.*, Transmission of SARS-CoV-2 in Domestic Cats. *N. Engl. J. Med.* (2020) https:/doi.org/10.1056/NEJMc2013400.

9. M. Hoffmann, *et al.*, SARS-CoV-2 Cell Entry Depends on ACE2 and TMPRSS2 and Is Blocked

by a Clinically Proven Protease Inhibitor. *Cell* (2020) https:/doi.org/10.1016/j.cell.2020.02.052.

10. B. Rockx, *et al.*, Comparative pathogenesis of COVID-19, MERS, and SARS in a nonhuman primate model. *Science* (2020) https:/doi.org/10.1126/science.abb7314.

11. J. Shi, *et al.*, Susceptibility of ferrets, cats, dogs, and other domesticated animals to SARS-coronavirus 2. *Science* (2020) https:/doi.org/10.1126/science.abb7015.

12. T. H. C. Sit, *et al.*, Infection of dogs with SARS-CoV-2. *Nature* (2020) https:/doi.org/10.1038/s41586-020-2334-5.

13. M. Wang, *et al.*, Remdesivir and chloroquine effectively inhibit the recently emerged novel coronavirus (2019-nCoV) in vitro. *Cell Res.* **30**, 269–271 (2020).

14. J. Harcourt, *et al.*, Isolation and characterization of SARS-CoV-2 from the first US COVID-19 patient. *bioRxiv*, 2020.03.02.972935 (2020).

15. V. J. Munster, *et al.*, Respiratory disease and virus shedding in rhesus macaques inoculated with SARS-CoV-2. *bioRxiv*, 2020.03.21.001628 (2020).

16. K. Schlottau, *et al.*, Experimental Transmission Studies of SARS-CoV-2 in Fruit Bats, Ferrets, Pigs and Chickens. *SSRN* (2020) https:/doi.org/10.2139/ssrn.3578792 (May 16, 2020).

17. C. Shan, *et al.*, Infection with Novel Coronavirus (SARS-CoV-2) Causes Pneumonia in the Rhesus Macaques. *Research Square* (2020) https:/doi.org/10.21203/rs.2.25200/v1.
